# Wuho Is a New Member in Maintaining Genome Stability through its Interaction with Flap Endonuclease 1

**DOI:** 10.1371/journal.pbio.1002349

**Published:** 2016-01-11

**Authors:** I-Cheng Cheng, Betty Chamay Chen, Hung-Hsun Shuai, Fan-Ching Chien, Peilin Chen, Tao-shih Hsieh

**Affiliations:** 1 Institute of Cellular and Organismic Biology, Academia Sinica, Taipei, Taiwan; 2 Department of Optics and Photonics, National Central University, Chung-Li, Taiwan; 3 Research Center for Applied Sciences, Academia Sinica, Taipei, Taiwan; 4 Department of Biochemistry, Duke University, Durham, North Carolina, United States of America; National Cancer Institute, UNITED STATES

## Abstract

Replication forks are vulnerable to wayward nuclease activities. We report here our discovery of a new member in guarding genome stability at replication forks. We previously isolated a *Drosophila* mutation, *wuho* (*wh*, no progeny), characterized by a severe fertility defect and affecting expression of a protein (WH) in a family of conserved proteins with multiple WD40 repeats. Knockdown of WH by siRNA in *Drosophila*, mouse, and human cultured cells results in DNA damage with strand breaks and apoptosis through ATM/Chk2/p53 signaling pathway. Mice with m*Wh* knockout are early embryonic lethal and display DNA damage. We identify that the flap endonuclease 1 (FEN1) is one of the interacting proteins. Fluorescence microscopy showed the localization of WH at the site of nascent DNA synthesis along with other replication proteins, including FEN1 and PCNA. We show that WH is able to modulate FEN1’s endonucleolytic activities depending on the substrate DNA structure. The stimulatory or inhibitory effects of WH on FEN1’s flap versus gap endonuclease activities are consistent with the proposed WH’s functions in protecting the integrity of replication fork. These results suggest that *wh* is a new member of the guardians of genome stability because it regulates FEN1’s potential DNA cleavage threat near the site of replication.

## Abstract 2

The essential protein Wuho can interact with FEN1 and switch its activity from potentially damaging gap endonuclease to useful flap endonuclease, thereby helping to protect the integrity of the replication fork and maintain genome stability.

## Author Summary

Accurate genome replication is essential for the transmission of genetic information, and this process is vulnerable to factors that can induce DNA damage or block replication. To avoid this and achieve genome stability, many enzymes need to coordinate their activities at the replication forks (the area where the two DNA strands separate to replicate), thereby ensuring high efficiency and fidelity. Replication occurs simultaneously in both strands, but DNA polymerases only work in one direction (5′-to-3′), and while synthesis of one strand proceeds continuously, synthesis of the other must occur via small DNA fragments that need to be joined together after the removal of their 5′ ends by the flap endonuclease 1 (FEN1). In addition, FEN1 has a gap endonuclease activity that can potentially introduce strand breaks at the sites of replication. Here we report the identification of a new member of this complex of proteins called Wuho (WH) that has a role in guarding the genome stability at the replication forks. Down-regulation of Wuho in tissue culture cells results in double-strand DNA breaks and programmed cell death. Genetic and biochemical results indicate that WH interacts with FEN1 and regulates its activities at the replication fork by promoting its flap endonuclease activity while dampening its gap endonuclease activity. These results suggest a critical role of Wuho in protecting the integrity of replication forks.

## Introduction

Faithful DNA replication is important for both the proliferation of cells and transmission of genetic information. This key biological process is vulnerable to many impediments, including numerous intrinsic and extrinsic agents that can damage DNA and create blockage for the movement of enzymatic machinery at the replication forks. At this fork, there are two separate but well-coordinated DNA biosynthetic activities, one for each template strand of parental duplex. Because of the 5′-3′ unidirectional activity of DNA polymerases, the leading strand synthesis can proceed without any interruptions after the initiation, while the lagging strand synthesis is intermittently halted for multiple RNA priming events and results in generating discontinuous nascent DNA, the so-called Okazaki fragments [1,2]. The ligation of the lagging strands requires the removal of their 5′-RNA primers, and this remodeling process in eukaryotes is usually carried out by the flap endonuclease 1 (FEN1) [3–5].

The bacterial FEN1 homologue was characterized as an integral part of *Escherichia coli* DNA polymerase I [6,7]. In eukaryotic organisms, FEN1 was first cloned and characterized from mouse cells by Harrington and Lieber [8], and it forms a ubiquitous and conserved family of enzymes. FEN1 is a structure-specific endonuclease capable of removing DNA or RNA flaps branching out from duplex DNA [4,9]. In addition to its important role in remodeling nascent lagging strands prior to their ligation, FEN1 is involved in repairing DNA damage in the pathway of long-patch base-excision repair [10]. Interestingly, FEN1 is known to possess a gap endonuclease activity, which may be functional in genetic recombination but also poses a potential threat for replication forks [11]. The critical biological functions of FEN1 are evidenced by the observation that homozygous *fen1* knockout mice are early embryonic lethal [12]. The heterozygous *fen1* mutation also promotes rapid tumor progression in a cancer-prone mouse model [13]. FEN1 is known to be under both positive and negative regulations. There are a number of proteins shown to interact with FEN1, including PCNA, 9-1-1, Blm, and Wrn helicases, and they are able to stimulate the FEN1’s in vitro endonucleolytic activities [14–17]. FEN1 is under multiple post-translational modifications that can either inhibit its enzymatic activities or promote its degradation [18–20]. We describe here the discovery of a new FEN1-interacting protein, Wuho, which has an essential role in genome stability, possibly through its ability to modulate FEN1’s activities depending on the structure of the DNA substrate.

**Fig 1:**
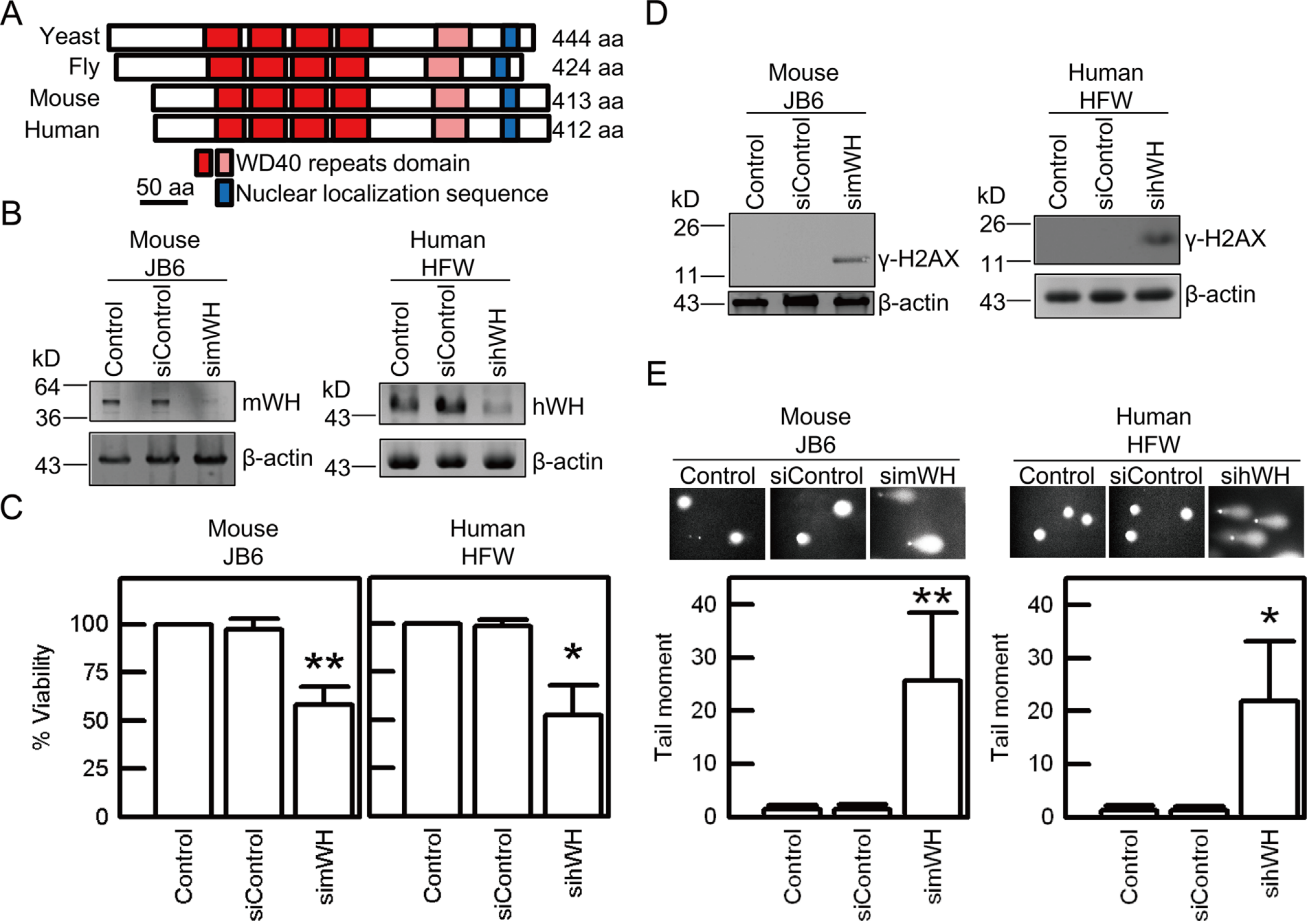
Depletion of Wuho protein (WH) induces loss of viability and DNA damage in mouse JB6 and human HFW cell lines. Wuho (WH) homologues display similarity in protein sequence and function of cellular protection. (A) Sequence conservation of Wuho shown by alignment of WH homologues in yeast (*Saccharomyces cerevisiae*), fly (*Drosophila melanogaster*), mouse (*Mus musculus*), and human (*Homo sapiens*), based on a previous study [21]. Dark and light red boxes represent WD40 repeats of high and low similarity, respectively, and blue boxes represent nuclear localization sequence. Scale bar is 50 amino acids. (B) Mouse Wuho protein (mWH) and human Wuho protein (hWH) were depleted by treatment of 25 nM of mWH siRNA (simWH) or hWH siRNA (sihWH) in cultured cells for 72 h. No changes in WH levels were observed in Control (cells without treatment) or siControl (cells treated with control siRNA) groups. (C) Depletion of WH causes loss of viability as determined by MTT assays. (D) Depletion of WH induces DNA damage revealed by γ-H2AX staining. (E) DNA damage monitored by neutral comet assay. The levels of DNA double strand breaks were quantified as tail moment (the product of tail intensity and tail length) shown at lower panels. Single asterisk and double asterisks indicate significant differences when compared with the control groups at *p* < 0.05 and *p* < 0.01 levels, respectively, according to Student’s *t* test.

We previously identified *wuho* (GeneID: 31566; protein accession: NP_572307.1) through isolating a *Drosophila* mutant deficient in its expression and demonstrated that it has a sterile phenotype (*wuho* means no progeny in Chinese, abbreviated as *wh*) [21]. Wuho (WH) belongs to an evolutionarily conserved family of proteins (Fig 1A), known as TRM82 in yeast (GeneID: 851743; protein accession: NP_010449.1) and WDR4 in humans (GeneID: 10785; protein accession: NP_387510.1) and mice (GeneID: 57773; protein accession: NP_067297.2). It is characterized by the presence of multiple WD40 repeats and can form a disc-like structure with seven β-propeller blades [22]. WD40 proteins are known for their function in mediating the formation of macromolecular complexes important for multiple cellular processes [23]. WH’s homologue in yeast, TRM82, is the non-catalytic subunit for heterodimeric m^7^G46 tRNA methyltransferase with the catalytic subunit known as TRM8 [24]. However, it is unclear that the tRNA methylase activity of TRM8/TRM82 complex has any essential cellular functions since mutations in either gene do not affect yeast viability [24]. On the other hand, our work here demonstrates that *wh* has an essential function in mice and that it has a conserved critical role in maintaining genome stability in metazoans, likely through WH’s function of regulating FEN1’s enzymatic activities.

## Results

### *wh* Contributes to Genome Stability

In *Drosophila*, *wh* has an important function in the growth and differentiation of germline cells [21]. To probe the cellular functions of *wh* in mammalian cells, we utilized small interference-RNA (siRNA) to knockdown WH expression in mouse JB6 and human HFW cells. Compared with control cells and cells treated with non-targeting control RNA, siRNA specific for *wh* can reduce WH expression in both JB6 and HFW cells (Fig 1B). Depletion of mouse WH (mWH) by simWH and human WH (hWH) by sihWH results in greatly reduced viability of JB6 and HFW, respectively (Fig 1C). The loss of viability is likely due to a failure in maintaining genome stability. The depletion of WH induces DNA strand breaks as shown by γ-H2AX signals detected by western blot (Fig 1D) and also by comet assay in a neutral buffer aiming to detect double strand breaks (Fig 1E). We probed the biological functions of *wh* in *Drosophila* S2 cells and showed that WH knockdown results in loss of viability, DNA damage, and apoptosis (S1 Fig). These experiments thus demonstrate that *wh* has an evolutionarily conserved function, in the metazoan cultured cells, to maintain genome stability.

**Fig 2:**
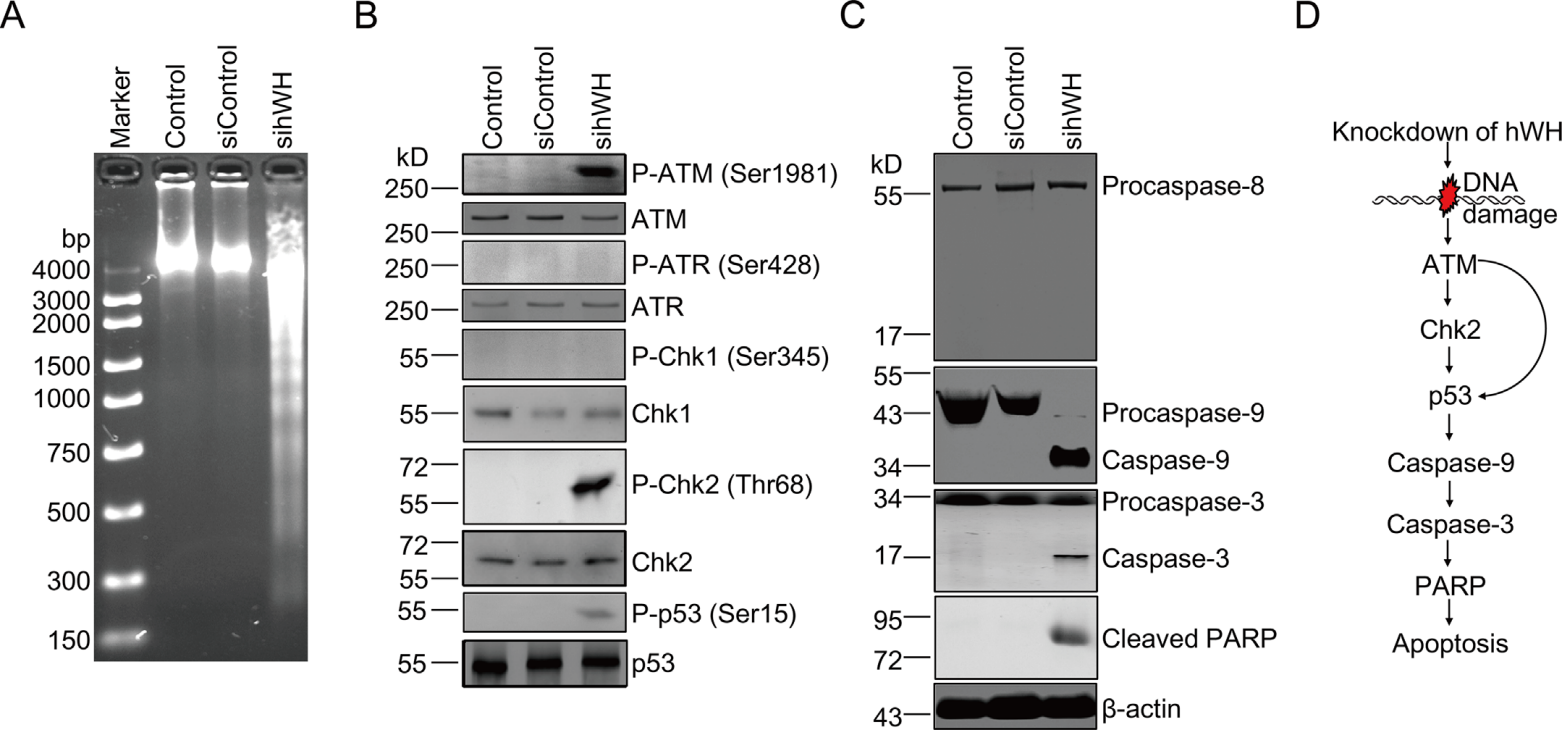
Signaling path of cell death following depletion of hWH in human HFW cells. (A) DNA laddering reveals apoptosis in cells with hWH knockdown. (B) Activation of key signaling molecules by phosphorylation ensuing hWH knockdown. Activation of DNA damage signaling pathway was through phosphorylation of ATM (Ser1981), Chk2 (Thr68), and p53 (Ser15), but not through phosphorylation of ATR (Ser428) and Chk1 (Ser345). (C) Apoptosis was driven by an intrinsic but not extrinsic pathway. Activation of Caspases was monitored by the cleavage of Procaspase-9, Procaspase-3, and PARP, but not Procaspase-8. (D) Diagrammatic summary of DNA damage signaling and apoptotic pathway after depletion of hWH.

To demonstrate the relevance of DNA damage induced by the depletion of WH to the loss of viability, we examined whether these cells were affected by apoptosis. By agarose gel electrophoresis of the isolated genomic DNA, we showed that depletion of hWH results in degradation of nuclear DNA in HFW cells and the generation of the characteristic fragmentation pattern of DNA ladders (Fig 2A).

We investigated the signaling pathway connecting DNA strand breaks to apoptosis after hWH knockdown. An initial signaling event due to double-strand breaks is expected to involve the activation of the ATM/Chk2 pathway [25,26]. This is confirmed by our observation that ATM activation through Ser1981 phosphorylation and Chk2 phosphorylation at Thr68 occurred following hWH depletion, while the ATR/Chk1 pathway was not affected (Fig 2B). A key regulatory molecule, p53, coordinates DNA damage signaling and subsequent cell cycle arrest and apoptosis [27]. hWH knockdown leads to the phosphorylation of Ser15 in p53, which is known to play a critical role in its pro-apoptotic activity [28,29].

Activation of p53 can lead to apoptosis through both transcription-dependent and–independent pathways to trigger the release of the Bcl2 family of proteins from mitochondria [30–32]. The subsequent release of cytochrome c can lead to the activation of Caspase-9 and the downstream Caspase-3 and cleavage of PARP (reviewed by [33,34]). The activation of the mitochondria-based Caspase-9 pathway was observed following hWH depletion, while the alternative Caspase-8 pathway was not activated (Fig 2C).

The notion that hWH has a critical function in preserving genome stability and cell survival is thus supported by the above data that depletion of WH leads to DNA strand breaks, ATM/Chk2/p53 activation and Caspase-9 initiated cell death (Fig 2D). Such a function of hWH is not restricted to human cells only; we have used mouse JB6 cells to demonstrate that the knockdown of mouse WH (mWH) results in the same pathway of DNA damage signaling and cell death (S2 Fig), which suggests a conserved function of WH in mammalian cells.

**Fig 3:**
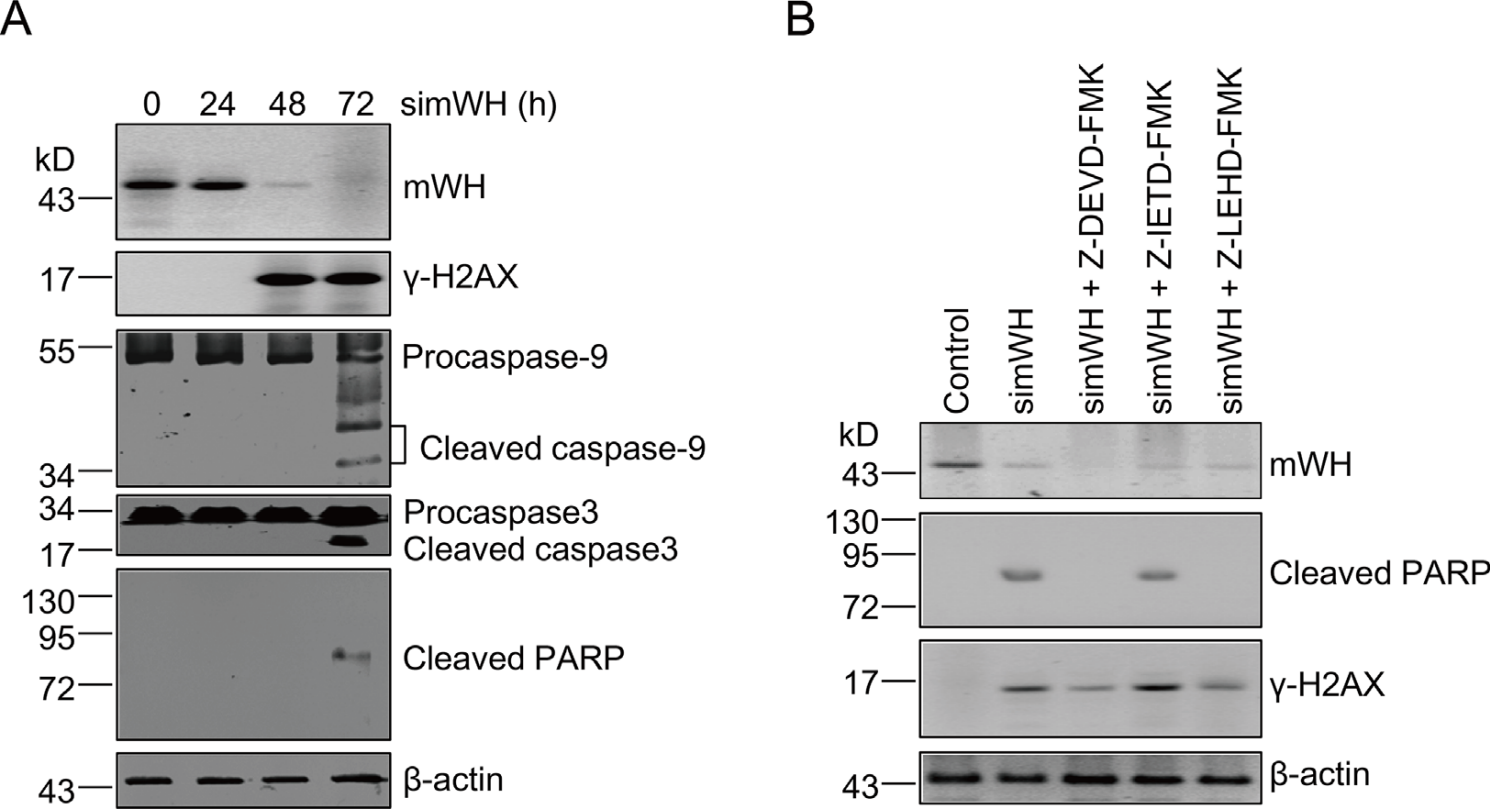
DNA damage precedes apoptosis after mWH depletion in mouse JB6 cells. (A) Time course of DNA damage and apoptosis following mWH depletion. Samples were collected for analysis following mWH knockdown at 0, 24, 48, and 72 h. DNA damage (γ-H2AX signals) was detected at 48 h after siRNA treatment. Apoptotic signals including cleavage of Procaspase-9, Procaspase-3, and PARP, were detected only after 72 h of treatment. (B) Inhibitors of Caspases-9 and -3, but not for Caspase-8, block apoptosis ensuing depletion of mWH. However, DNA damage signals were unaffected and present under these conditions. Cells were co-cultured with simWH and different Caspase-specific inhibitors (final concentrations of 2 μM in the culture medium) including Z-DEVD-FMK (Caspase-3), Z-IETD-FMK (Caspase-8), and Z-LEHD-FMK (Caspase-9). After 72 h of treatment, samples were collected and processed for western blot.

The results presented so far have showed that WH depletion resulted in DNA damage and cell death, but the order of these two events was not addressed here. It is possible that WH knockdown can promote apoptosis, and that DNA fragmentation associated with programmed cell death generates the damage signals observed here. To investigate the direct consequence of the removal of WH, we examined both the time course of appearances of key molecules and the effects of specific Caspase inhibitors on these molecules. Following the treatment of mWH siRNA (simWH), γ-H2AX appears before the activated Caspases and the cleavage product of PARP (Fig 3A), suggesting that DNA damage precedes the cell death program. This notion is further reinforced by results from treatment with Caspase-specific inhibitors. If apoptosis induces DNA damage signaling, Caspase inhibition should stall the appearance of γ-H2AX. With the addition of inhibitors for Capsase-8, -9, or -3 (Z-IETD-FMK, Z-LEHD-FMK, and Z-DEVD-FMK, respectively), the level of γ-H2AX was unaffected (Fig 3B). It is interesting to note that inhibition of Caspase-9 or -3 extinguishes the apoptosis response (no cleavage of PARP), while the addition of the Caspase-8 inhibitor has no effect, further confirming that apoptosis goes through the Caspases 9 and 3 pathway (see Fig 2D). These results suggest that WH plays an important role in genome stability and that its depletion leads to DNA breaks and cell death.

### p53 Plays a Critical Role in Apoptosis Driven by Depletion of hWH

**Fig 4:**
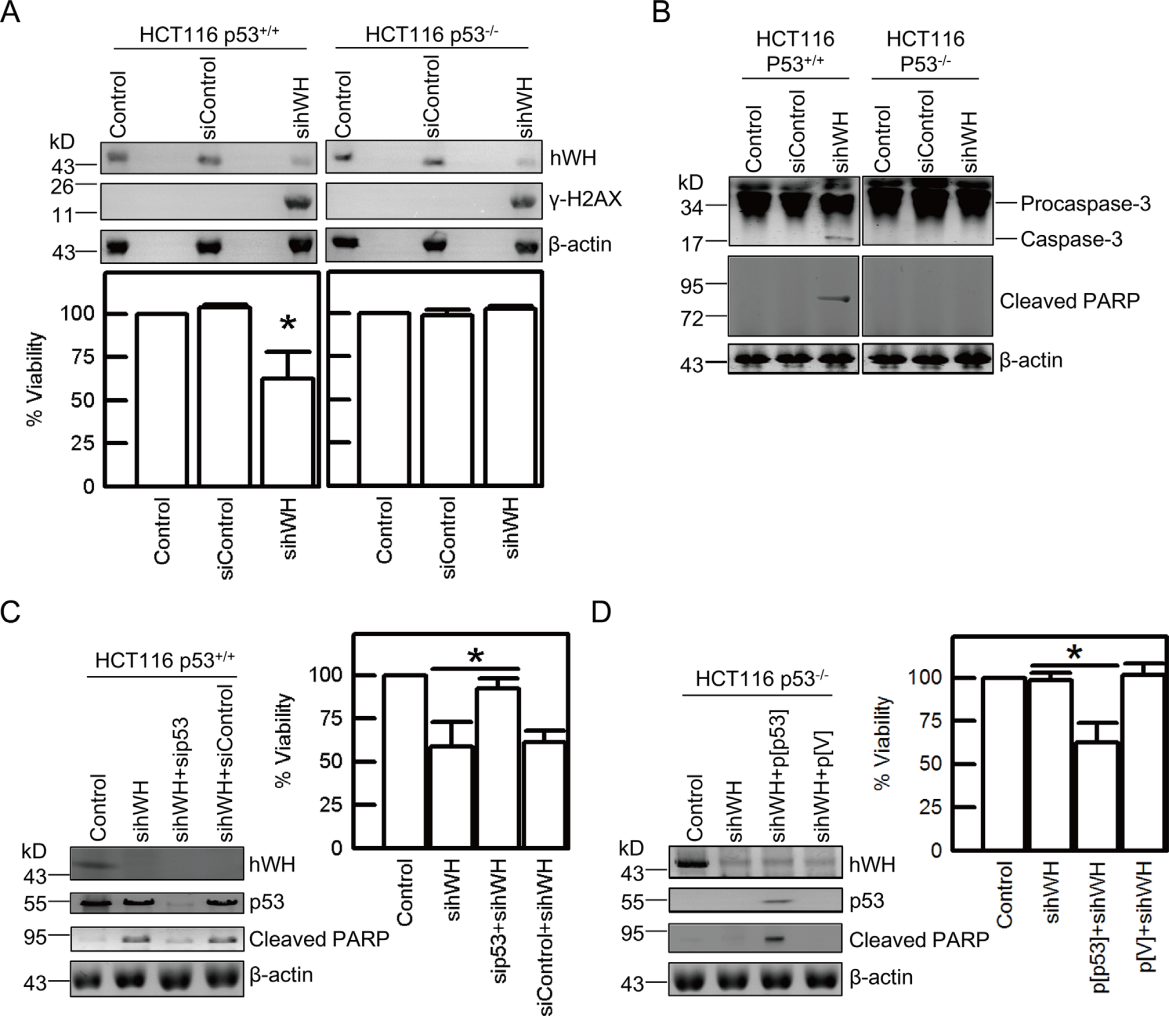
p53 plays a vital role in cell death following hWH depletion. (A) Depletion of hWH induces DNA damage (appearance of γ-H2AX) in both HCT116 p53^+/+^ and HCT116 p53^-/-^ cells, but only causes loss of viability in HCT116 p53^+/+^ cells. (B) Depletion of hWH causes apoptosis in HCT116 p53^+/+^ cells (revealed by the cleavage of Procaspase-3 and PARP), but not in HCT116 p53^-/-^ cells. (C) Knockdown of p53 mitigates apoptosis and improves viability after depleting hWH in HCT116 p53^+/+^ cells. Simultaneously knocking down hWH and p53 (by p53 siRNA, sip53) reduces PARP cleavage and increases cell viability in comparison with single knockdown of hWH. (D) Transfection of ectopic p53 in HCT116 p53^-/-^ cells promotes cell death resulting from depletion of hWH. Reversion of p53 status in HCT116 p53^-/-^ cells was carried out by transfection of pcDNA3.1/p53 construct (p[p53]). The expression of p53 protein was observed in p[p53] cells but not in vector control (p[V]). Single asterisk indicates significant difference at *p* < 0.05, according to Student’s *t* test.

Since the tumor suppressor protein p53 has a key role as a gatekeeper in maintaining genome stability by regulating the choice between cell death and cell cycle arrest upon genome damage (reviewed by [35]), we were interested in testing whether p53 plays a central role in directing the hWH-knockdown cells to apoptosis. We used a pair of isogenic cell lines with different p53 status, HCT116 p53^+/+^ and HCT116 p53^-/-^ [36], to test their response following hWH knockdown. Treatment with sihWH results in a similar reduction of hWH by 80% in both cell lines and induces DNA breaks as evidenced by appearance of γ-H2AX (Fig 4A). But only the cells with wild-type p53 status showed reduced cell viability (Fig 4A), and this was linked to the appearance of apoptotic markers including cleaved Caspase-3 and PARP (Fig 4B). To further confirm that their difference in entering apoptotic program is primarily determined by p53 status, we reversed p53 status by using siRNA knockdown in HCT116 p53^+/+^, and introduced a DNA vector for ectopic p53 expression in HCT116 p53^-/-^. After the manipulation to alter p53 status, we observed that the appearance of apoptotic markers and reduction in viability were reversed consequentially (Fig 4C and 4D). These results plus those showing p53 activation following hWH depletion suggest that hWH affects genome stability and that p53 has a key role in determining the cellular response to the genotoxic consequence resulting from hWH knockdown.

### m*Wh* Knockout Mouse Is Early Embryonic Lethal

**Fig 5:**
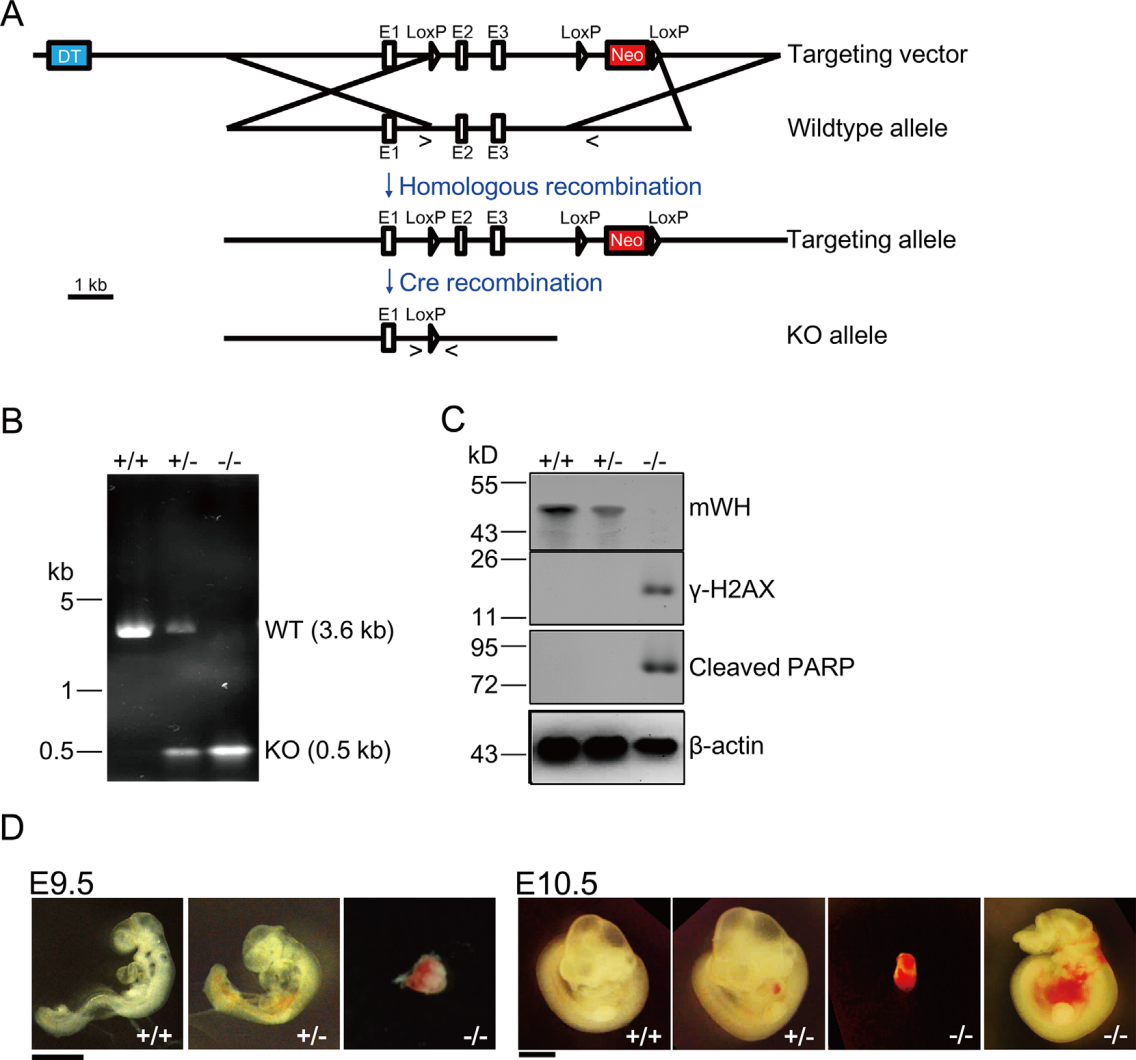
Mouse *Wuho* (m*Wh*) is an essential gene. (A) Gene targeting strategy. The gene targeting vector is a construct harboring positive (neomycin resistant gene [Neo], *red*) and negative (diphtheria toxin gene [DT], *blue*) selection markers. Through homologous recombination, the genomic region with exons 2 (E2) and 3 (E3) of m*Wh* was replaced and resulted in the introduction of one loxP site before E2, and the Neo cassette flanked by two loxP sites in Intron 3. Subsequently, E2 and E3 were deleted by Cre recombinase through recombination between two distal loxP’s. The symbols “>” and “<” represent the locations of forward and reverse primers used in PCR for genotyping. The PCR products are 3.6 kb from in wild type allele and 0.5 kb from knockout allele. (B) A representative genotyping experiment for embryos of E9.5 from heterozygous parents. PCR products can distinguish among genotypes of +/+, +/-, and -/-. (C) Knockout of m*Wh* results in DNA damage and apoptosis. The embryos genotyped in (B) were processed for western blot to examine expression of mWH, appearance of γ-H2AX for DNA damage, Cleaved PARP for apoptosis, and actin as loading controls. (D) Development of -/- embryos at E9.5 (left panel) and 10.5 (right panel) is abnormal. In E10.5 -/- embryos, they show varying morphology, potentially due to different degrees of resorption. Severe extents of resorption and are small (the left one of -/-). The other null embryo (the right one of -/-) show minor morphological defects and has brain development defects and internal bleeding. Scale bar is 1 mm.

Given that siRNA knockdown experiments suggest that *wh* has a critical function in genome maintenance and cell growth in mammalian cells, we sought to test its function in an organismic context. We constructed a knockout vector to delete the second and third exons in the m*Wh* gene (Fig 5A). We examined 110 progeny from a cross of heterozygous m*Wh* knockouts and found no pups that were homozygous m*Wh* nulls (Table 1). But the pups of the heterozygous mutant and those of the homozygous wild type followed an expected 2:1 ratio. Upon dissecting the ovaries of pregnant females, we could identify m*Wh* null by genotyping with PCR (Fig 5B), and these embryos can be detected up to day 10.5 (Table 1). Western blot experiments using embryo samples showed that mWH is depleted in the null and the heterozygous has about half of the wild-type amount (Fig 5C). Interestingly, we could detect both DNA damage and apoptotic signals, γ-H2AX and cleaved PARP, in m*WH* null embryos (Fig 5C). This suggests the lethality of mouse embryos follows a similar pathway to that of cultured cell lines with WH-knockdown. In addition, some of the null embryos at day 9.5 showed a resorbed morphology. There are apparent morphological differences in null embryos at day 10.5 and they showed varying aberrations, possibly due to different degrees of resorption. E10.5 nulls can be severely resorbed or have abnormality of brain development and internal bleeding (Fig 5D). The genetic experiments with *Drosophila* and mice, as well as the siRNA knockdowns with tissue culture cells, demonstrated the critical role of *wh* in cell growth and development, possibly through involvement in maintaining genome stability.

**Table 1:** Genotypes of embryos and progeny from *wuho* heterozygous intercrosses.

Age	Number with genotype			Total number of mice
	+/+	+/-	-/-	
E7.5	2	4	3	9
E8.5	1	3	2	6
E9.5	12	11	5*	28
E10.5	6	7	3*	16
E11.5	4	5	0	9
E16.5	2	3	0	5
E17.5	1	5	0	6
E18.5	3	7	0	10
Live born	37	73	0	110

*Abnormal embryos

### hWH’s Interacting Partners Include a Flap Endonuclease FEN1

**Fig 6:**
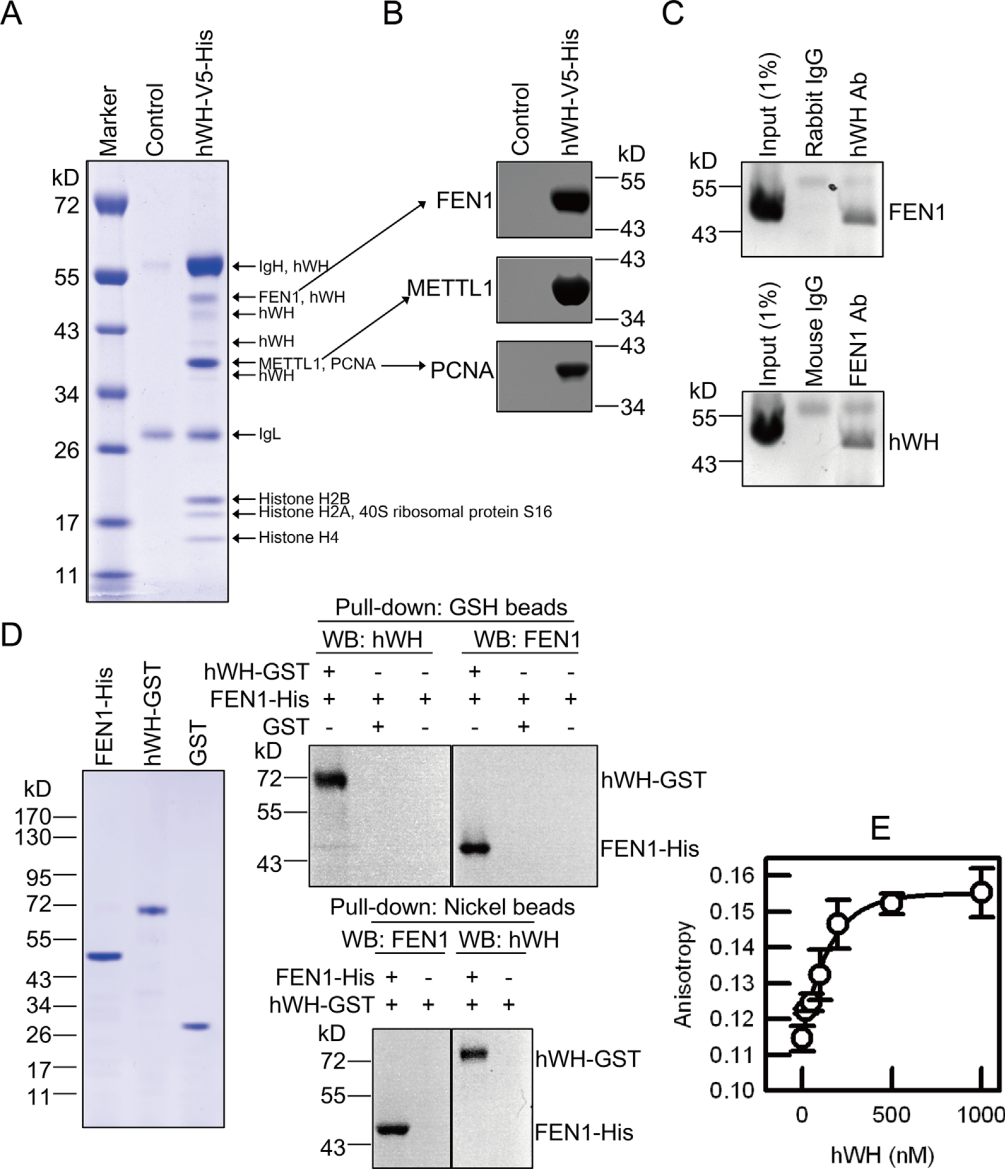
hWH physically interacts with FEN1. (A) Identification of proteins present in hWH immunoprecipitate. We induced hWH expression in cells with an ectopic expression vector of hWH with V5 and His tags. The cleared cell lysate was incubated with anti-V5 agarose beads. The bead-bound proteins were separated by SDS-PAGE and stained with Coomassie blue. Each band was sliced and identified by Mass Spectrometry. The cells transfected with empty vector served as a control. (B) Flap endonuclease 1 (FEN1), methyltransferase-like protein 1 (METTL1), and proliferating cell nuclear antigen (PCNA), three candidates identified in (A) were further confirmed with western blot. (C) Interaction between endogenous hWH and FEN1 was demonstrated by co-IP experiments using cell lysate. hWH or FEN1 from cell lysate was removed by Protein A beads by using the antibody against hWH or FEN1, followed by probing the partner protein with western blot. The bands above both hWH and FEN1 are IgG heavy chains. (D) Direct interaction between hWH and FEN1 demonstrated by pull-down assays. FEN1 tagged with C-terminal His_6_, hWH tagged with C-terminal GST, and GST proteins were purified, and examined by SDS-PAGE and staining with Coomassie blue to verify their purity (left panel). hWH tagged with C-terminal GST and FEN1 tagged with C-terminal His_6_ were incubated with either Glutathione beads or Ni(II)-NTA beads, followed by detecting the association of partner proteins with western blot (right panels). Negative controls were beads alone without baits. (E) Direct binding between hWH and FEN1 revealed by fluorescence anisotropy. Tetracysteine-tagged FEN1 (FEN1-CCPGCC) was labeled with ReAsH fluorophore. Fluorescence anisotropy was measured after adding varying concentrations (0–1,000 nM) of hWH to 50 nM ReAsH-labeled FEN1-CCPGCC. The data points were used to fit a binding isotherm with a dissociation constant of 130 nM.

To probe the molecular mechanism by which hWH participates in maintaining genome stability, we sought to determine its interacting partners by co-immunoprecipitation (coIP) and analysis with mass spectrometry. From a human 293 cell line with inducible expression of hWH tagged with V5 and hexahistidine epitopes, we isolated the nuclear extract and incubated it with agarose beads conjugated with V5 monoclonal antibody. The bead-bound proteins were eluted, resolved by polyacrylamide gel electrophoresis, and analyzed by Mass Spectrometry (Fig 6A). Three of the identified proteins may provide information regarding hWH’s functions: METTL1 (Methyl Transferase-like Protein 1, human homolog of the yeast tRNA methyltrasferase subunit TRM8), FEN1, and PCNA. The identity of these proteins was also confirmed by western blot of the bead-bound proteins (Fig 6B).

The WH homolog in yeast is TRM82, the non-catalytic subunit of tRNA methyltransferase, that forms a heterodimer with the catalytic subunit TRM8 [24]. Human homologs of TRM8 and TRM82 can form a complex that has tRNA methyltransferase activity [24,37]. However, neither TRM8 nor TRM82 is essential for yeast growth [24,38]. Results presented in the next section also suggest that tRNA methyl transferase activity is not relevant to genome instability when hWH is depleted.

FEN1 and PCNA can form a complex and function at the replication fork to remove the RNA primers and remodel Okazaki fragments for facilitating the religation of the lagging strands [5]. Since earlier research had no indications that either one can interact with WH, we sought to establish their biochemical interactions. Whereas hWH partners were identified here through coIP with an ectopically expressed and tagged hWH, we carried out coIP experiments with endogenous hWH and FEN1 from HCT116 p53^+/+^ cells to verify their interactions (Fig 6C). These results demonstrate that hWH/FEN1 interaction does not depend on the presence of an ectopically expressed protein with an epitope tag. To examine if the binding is through direct protein/protein interaction, we purified recombinant proteins of hWH tagged with GST and FEN1 with hexahistidine and used GSH beads and Ni(II)-NTA resin, respectively, to pull down their partners (Fig 6D). These results demonstrate that hWH and FEN1 can have direct protein/protein interaction without the need of a mediator partner. As an alternative approach to directly demonstrate the interaction between FEN1 and hWH, we monitored the fluorescence anisotropy change of a fluorophore-tagged hFEN1 due to its association with hWH (Fig 6E). The increase in anisotropy depends on the addition of hWH and is saturable at higher hWH concentrations, confirming that a specific linkage between FEN1 and hWH results in a change in rotational diffusion time and a higher anisotropy for the tagged protein. The anisotropy data can be used to determine a dissociation constant of 130 nM.

Similar pull-down experiments with purified recombinant hWH and PCNA, however, failed to demonstrate their interactions (S3A and S3B Fig). But FEN1 is known to interact with PCNA [39], and the interaction of PCNA with FEN1 and many other replication proteins is through the PIP (PCNA-Interacting Protein) motif [40]. Since most of the PCNA partners possess the PIP motif [41], and hWH does not have one, it is not surprising that we could not observe a direct interaction between hWH and PCNA. It is possible that hWH associates with FEN1/PCNA complex through its binding to FEN1, and functions at the replication forks in facilitating the maturation of Okazaki fragments. Indeed, we can demonstrate that the pull-down of PCNA by hWH only occurs in the presence of FEN1 (S3B Fig).

### hWH Is Localized at the DNA Replication Site, Along with FEN1 and PCNA

**Fig 7:**
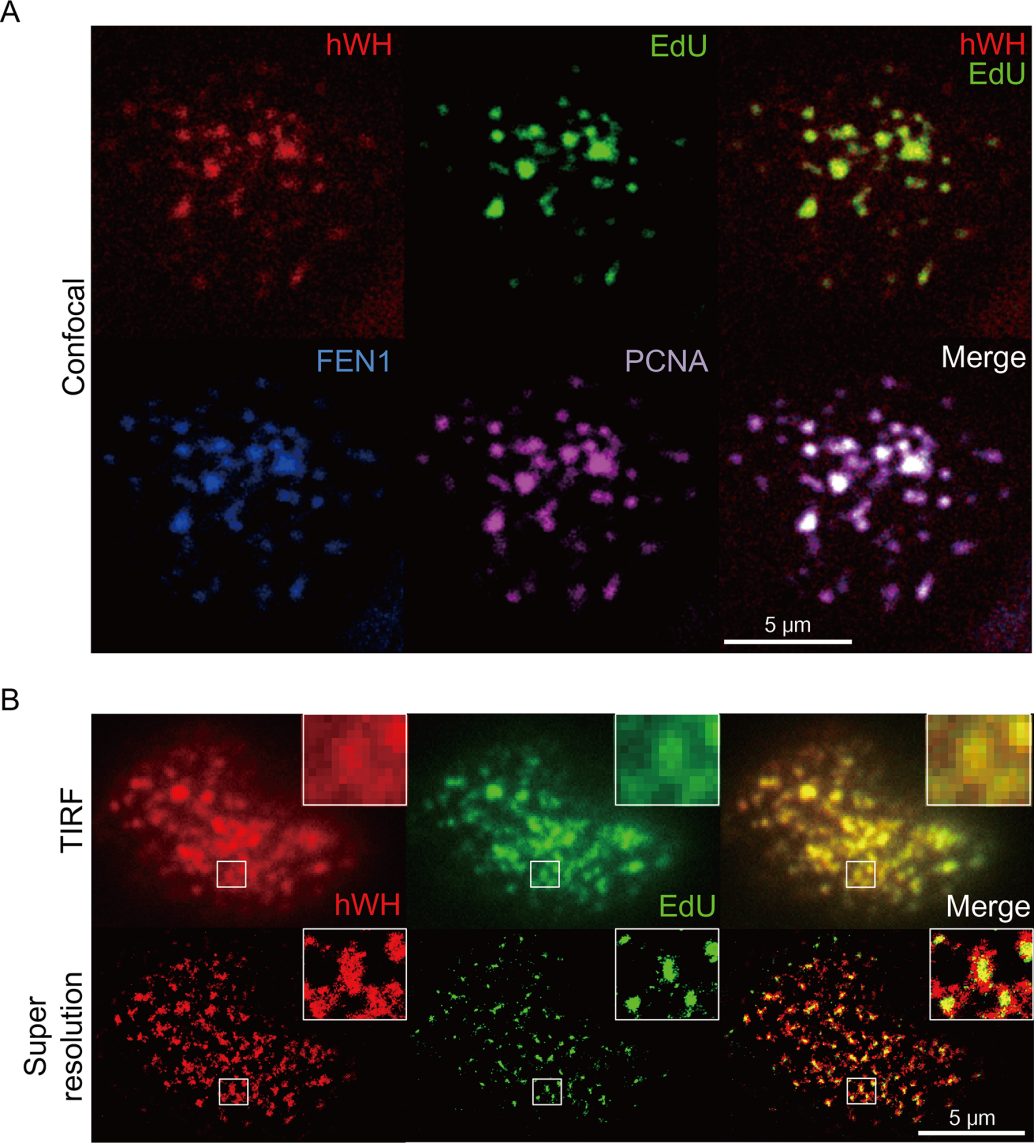
Localization of hWH at the site of DNA replication determined by confocal (A) and super-resolution microscopy (B). (A) HCT116 p53^+/+^ cells were stained with rabbit anti-hWH antibody (red), mouse anti-FEN1 antibody (blue), goat anti-PCNA (purple), and the nascent DNA replication marker EdU (green). The merged images of hWH plus EdU, or all four signals, are in the upper-right and lower-right panels, respectively. (B) HCT116 p53^+/+^ cells were stained with rabbit anti-hWH antibody (red) and EdU (green). The images were obtained by TIRF microscopy and super-resolution microscopy. The boxed areas are shown at higher magnification.

To further probe hWH’s function in genome stability, we investigated its intracellular localization, especially with respect to the sites where DNA synthesis was ongoing. Both FEN1 and PCNA are localized at the site of newly synthesized DNA [42]. We therefore stained HCT116 p53^+/+^ cells for hWH, FEN1, PCNA, and EdU (5-ethynyl-2′-deoxyuridine), a thymidine analog, marking nascent nucleotide incorporation during DNA replication (Fig 7A). There were over 95% overlaps of the pixels from the images of FEN1, PCNA, and EdU when compared with hWH. The results showed that these three proteins were co-localized with DNA replication sites, suggesting that hWH is involved in DNA replication.

Besides confocal fluorescence microscopy, we also utilized a super-resolution imaging technique dSTORM, direct stochastic optical reconstruction microscopy [43], to verify the co-localization pattern between hWH and EdU. The dSTORM system is based on total internal reflection fluorescence (TIRF) microscopy. Therefore, we can obtain both TIRF and dSTORM (super-resolution) images from the same sample, reinforcing the validity of dSTORM result (Fig 7B, upper and lower panels, respectively). The TIRF and dSTORM images demonstrated co-localization of hWH and EdU. The higher magnification inserts of these images further support the co-localization pattern even at the super-resolution level (Fig 7B, lower panels). The localization of Wuho at replication loci is also shown recently with iPOND using the method of chemical cross-linking and proteomic analysis (listed as WDR4 in Table S6 in [44])

**Fig 8:**
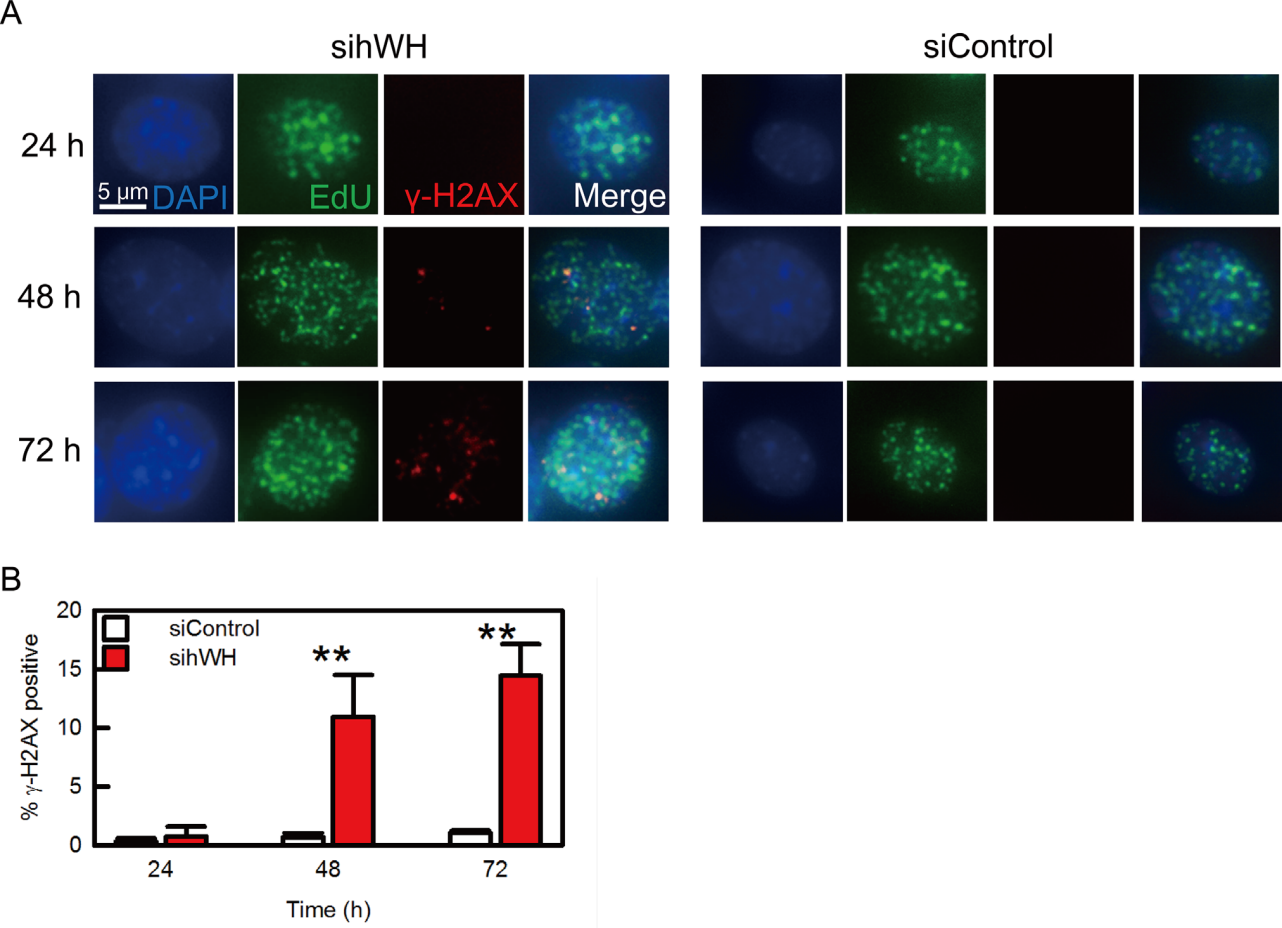
hWH knockdown results in DNA damage at the replication sites. (A) HCT116 p53^+/+^ cells were treated with either hWH or control siRNA for 24, 48, and 72 h. Afterward cells are harvested and stained with DAPI (blue), EdU (green), and γ-H2AX (red). (B) After siRNA treatment for 48h, γ-H2AX positive cells percentage in sihWH is significantly higher than that of siControl. Double asterisks indicate significant differences at *p* < 0.01, according to Student’s *t* test.

The notion that hWH has a function to protect DNA integrity at the replication fork predicts that upon hWH knockdown, DNA damage should occur at the site of replication. We tested this hypothesis by monitoring the localizations of replication and damage sites marked by EdU and γ-H2AX, respectively, during the time course following hWH knockdown (Fig 8). We observed that γ-H2AX staining colocalizes with EdU (the overlap of EdU to γ-H2AX is 92.9%, and the reciprocal of γ-H2AX/EdU is 5.9%), consistent with the idea that hWH’s genome stability function is at replication sites (Fig 8A). The γ-H2AX staining begins to appear at 48-h after hWH siRNA treatment, and it persists to 72-h (Fig 8B).

To further verify the role of hWH in protecting replication fork integrity, rather than having a direct role in DNA repair, we treated HCT116 p53^+/+^ cells with X-ray-irradiation and monitored hWH’s expression levels and nuclear distribution. We observed appearance of the DNA damage marker γ-H2AX in a time and dose dependent manner but with hWH’s expression levels remaining unchanged (S4A Fig). We also examined hWH’s intracellular localization to investigate whether hWH colocalizes with DNA damage loci. Immunostaining results show that hWH’s localization is independent of irradiation-induced γ-H2AX signals (S4B Fig). These results thus show that Wuho localizes at and protects replication forks, but it is not directly linked to DNA repair process.

### hWH’s Function in Genome Stability Is through the Action of FEN1

**Fig 9:**
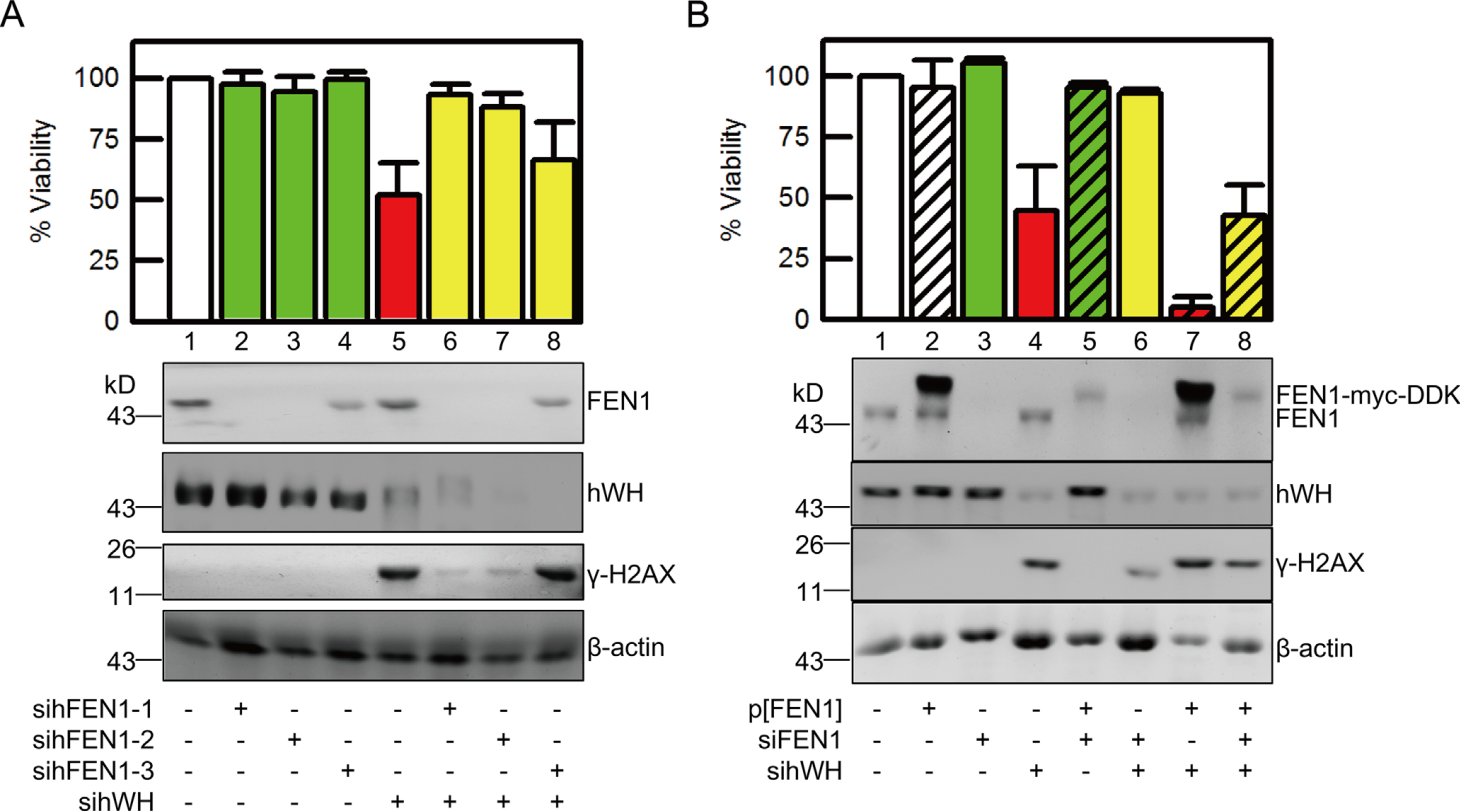
FEN1 plays a vital role in DNA damage and cell death induced by knockdown of hWH. (A) Depletion of FEN1 mitigates DNA damage and cell death resulting from hWH knockdown in human cells. Double knockdowns of hWH and FEN1 were conducted in HCT116 p53^+/+^ cells by siRNAs. We utilized the siRNA pool to knockdown hWH, and three different single siRNAs for FEN1 knockdown. The depletion efficiency of hWH and FEN1, and the DNA damage level revealed by γ-H2AX were determined by western blot. Viability of each treatment was measured by MTT assay. Two of the FEN1 siRNA showing better FEN1 knockdown efficiency (sihFEN1-1 and -2, lanes 2 and 3) were able to better rescue DNA damage and cell death induced by hWH knockdown (lanes 6 and 7). (B) Ectopic expression of FEN1 reverses the rescuing effect by FEN1 knockdown and makes hWH knockdown more toxic. Transfection of vector for ectopic expression of FEN1 tagged with myc and DDK was conducted with pCMV6/FEN1-myc-DDK construct (0.2 μg/ml for 10^5^ cells). Knockdowns of hWH and FEN1 were carried out with siRNA pools. While knockdown of FEN1 can rescue cytotoxicity induced by hWH knockdown (lanes 4 versus 6), ectopic expression of FEN1 can restore the cytotoxicity of hWH knockdown (lanes 6 versus 8). Interestingly, combined expression of FEN1 from both endogenous and ectopic sources exacerbates cytotoxic effect of hWH depletion (lanes 4 versus 7).

The association of hWH with FEN1/PCNA, its localization at the site of DNA synthesis, and its knockdown resulting in DNA strand breaks suggest a plausible mode of action for hWH’s role in genome stability. The nuclease activity of FEN1 while necessary for removing RNA primers and DNA damage, also presents a possible threat to genome integrity especially near the replication forks. If hWH can modulate the nuclease activities of FEN1, it may thereby mitigate a potential hazard posed by FEN1. This would also have the implication that the DNA damage following the reduction of hWH expression is mediated through FEN1. We tested this hypothesis by showing that while knocking down FEN1 only minimally affects cell viability, double knockdown of hWH and FEN1 improves the viability in comparison with hWH knockdown alone (Fig 9A). Moreover, we tested three separate sihFEN1 knockdown siRNAs, and they all were able to ameliorate the viability loss due to hWH knockdown. The rescuing ability is correlated with the knockdown efficiency with the one less capable of reducing FEN1 expression (sihFEN1-3) having lower viability restoration (lanes 6 and 7 versus 8, Fig 9A). It is also interesting that double knockdown of hWH and FEN1 mitigates the DNA strand breaks or the levels of γ-H2AX relative to hWH knockdown alone (Fig 9A). This phenomenon of synthetic rescuing can be replicated in both human (Fig 9A) and mouse cells (S5 Fig). Interestingly, the rescuing effect by FEN1 knockdown can be reversed by ectopic expression of FEN1 (lanes 6 versus 8, Fig 9B). With FEN1 overexpression under the conditions of both ectopic and endogenous expression, knockdown of hWH becomes even more cytotoxic (lane 7). These data are consistent with the notion that hWH is able to modulate FEN1 nuclease activities and that a reduction in hWH’s expression presents a vulnerability for the unchecked nucleolytic action of FEN1.

Both our work and earlier work [24] suggest hWH has another interacting partner, METTL1, the catalytic subunit of tRNA methyltransferase. We therefore found it important to address whether METTL1 has a role in hWH’s genome stability. In contrast to hWH, knocking down METTL1 has no effects on DNA strand breaks or cell viability (S6A Fig). Furthermore, double knockdown of hWH and METTL1 does not improve either genome instability or cell viability relative to single hWH knockdown. That METTL1 is not relevant to genome stability is supported by its intracellular localization. In contrast to hWH, the nuclear localization of METTL1 is not coincident with loci of nascent DNA synthesis, as evidenced by confocal microscopy (S6B Fig). hWH thus has two intracellular functions, one associated with METTL1 as a partner for tRNA modification and the other involved in controlling FEN1 activities at the replication fork.

### Modulation of FEN1 Activities by hWH

**Fig 10:**
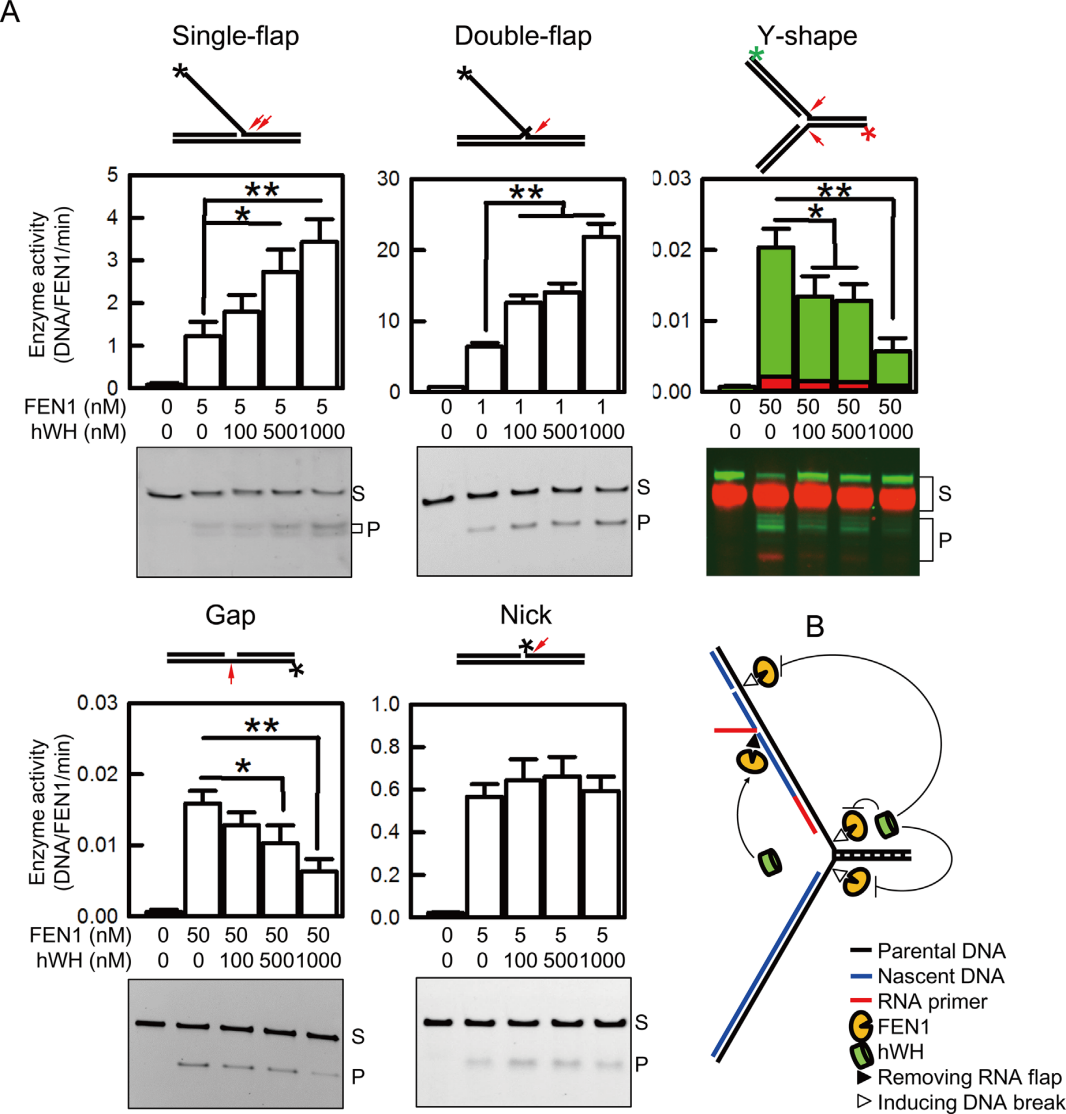
hWH modulating FEN1’s multiple nuclease activity suggests a protective role of hWH during DNA replication. (A) Structures of FAM-labeled substrates (single flap, double flap, gap, and nick) and FAM/Cy5 double labeled Y-shape (fork) substrate with cleavage sites marked by arrows are shown on top of each. FAM-labeling in each substrate is marked by an asterisk, and FAM- and Cy5-labeling in Y-shape substrate are marked by green and red asterisks, respectively. Results of activity assays examined by TBE-PAGE are shown beside their quantifications. The concentrations of DNA substrates were 50 nM, and those for FEN1 and hWH were indicated below. The bands indicated with letters S and P refer to substrate and product, respectively. Single asterisk and double asterisks indicate significant differences at *p* < 0.05 and *p* < 0.01 levels, respectively, according to Student’s *t* test. (B) Model for hWH regulating FEN1’s behavior near replication fork. hWH displays structure-specific modulation on FEN1 activity. hWH promotes FEN1’s flap endonuclease activity on RNA flap and inhibits the gap endonuclease activity on gap structure near lagging strand and replication fork. The sum of hWH action on FEN1 activity modulation suggests a protecting role of hWH near replication fork.

We employed biochemical approaches to directly test the hypothesis that hWH modulates FEN1’s nuclease activities. FEN1 can remodel Okazaki fragments through its 5′ endonuclease activities on either single or double flap substrates [5]. Interestingly, FEN1 possesses a low level of gap endonuclease activity at replication forks, a potentially hazardous activity for an enzyme located at the site of DNA synthesis [11]. FEN1’s gap endonuclease activity also can target double strand DNA with single strand region. Besides endonuclease activity, FEN1 has 5′ exonuclease ability. To test hWH’s effect on FEN1’s various nucleolytic functions, we generated five model substrates using fluorophore-tagged oligonucleotides and determined FEN1’s nuclease activities with and without hWH (Fig 10A). While hWH does not affect FEN1’s exonulease activity in nicked substrate, it exerts distinct effects on FEN1’s activities on the other DNA substrates. FEN1 displays the highest activity toward double flap substrate over other substrates, and for FEN1’s gap endonuclease activity on the Y-shape substrate, it is more active toward the lagging strand than the leading strand. These results are consistent with what were reported earlier [11,45,46]. Intriguingly, hWH stimulates FEN1’s flap endonuclease activity in single and double flap substrates but inhibits FEN1’s gap endonuclease in Y-shape and other gap substrates (Fig 10A). Either promotion or repression of these activities exhibits a dose dependence on hWH. These assays reported above were analyzed with DNA gel electrophoresis under denaturing conditions. We have also carried out assays using gap and Y-shape substrates but with samples processed for analysis using gel electrophoresis under non-denaturing conditions and obtained simialr results (S7 Fig). While the effects of hWH are most apparent at the highest concentration tested, 1 mM, representing a molar ratio in a range of 20- to 1,000-fold over FEN1, we could clearly observe statistically significant changes at much lower hWH concentrations as well. We could detect the stimulation of the flap endonuclease activity using single flap substrate with hWH at a 20-fold ratio over FEN1, and in a marked contrast, inhibition of gap endonuclease activity with Y-fork substrate at 2-fold molar ratio. These diametric effects of hWH on FEN1 depending the substrate structures are consistent with the biological functions of hWH proposed here.

While the biochemical basis for how hWH can regulate FEN1’s activities remains to elucidated, it is likely that the direct association of hWH with FEN1 as shown here affects its nucleolytic activities. But it remains possible that hWH can directly binds DNA with differential affinities. To test this possibility, we monitored binding of hWH with either double flap or Y-shape substrate with electrophoretic mobility shift assay. FEN1 can bind both DNA as reported in a previous study [45]. On the other hand, hWH does not show significant affinity with either (S8 Fig). Our results here suggest that hWH can interact with FEN1 and regulate its activities in a structure-specific manner, thereby providing a guardian role for FEN1’s biologically relevant functions at the replication fork (Fig 10B).

## Discussion

In *Drosophila*, *wh* mutations affect the growth and development of germline cells and result in male sterility and a greatly reduced fertility in female flies [21]. Because of maternal storage of WH protein in eggs, zygotic mutations of *wh* affect embryogenesis to a lesser extent, and some of the *wh* nulls can survive to adulthood. In an interesting contrast, we showed here that mouse homozygous nulls are embryonic lethal at days 9.5–10.5, demonstrating that the critical function of *wh* in cellular growth and development is evolutionarily conserved.

To further elucidate the mechanistic basis of *wh*’s essential functions, we developed a system for using siRNA to knockdown WH in tissue culture cells. WH knockdown in *Drosophila*, mouse, and human cells resulted in reduced viability and apoptosis. These cellular defects are due to double strand breaks, and through the signaling pathway of ATM/Chk2/p53 lead to Caspase-9-activated cell death. While programmed cell death will ultimately result in DNA fragmentation, our data showed that DNA cleavage due to WH knockdown is upstream to apoptosis, not caused by it. The time-course experiments following WH knockdown showed that DNA damage signals preceded Caspase activation. Using Caspase-specific peptide inhibitors, we showed that they mitigate Caspase cleavage/activation and PARP breakdown but not affecting DNA damage induced by WH-knockdown. While inhibitors for Caspases-9 and -3 blocked PARP cleavage, Caspase-8 inactivation had no effect on PARP processing. This result further supports the proposed pathway that apoptosis is mediated through Caspases-9 and -3, by the mitochondria-mediated intrinsic signaling mechanism, and not through the extrinsic signaling that would follow Caspase-8 activation. The apoptotic response emanating from genome instability is usually mediated through a p53-controlled and mitochondria-mediated pathway with ensuing activation of Capases-9 and -3 [33,34]. Therefore, WH-knockdown can lead to DNA strand breaks and result in programmed cell death through p53 activation.

To gain additional insight into the molecular mechanism by which WH can protect genome stability during the growth and development of an organism, we endeavored to examine its interacting partners by immunoprecipitation and Mass spectrometry. We identified a known interacting protein, METTL1, the catalytic subunit of tRNA methyl transferase, which heterodimerizes with hWH (also known as WDR4). However, it is unclear that WH’s function in genome stability is related to the activity of tRNA methyalse, since the yeast mutants defective in either subunits (mutations in TRM8 and TRM82) are viable and have no dramatic phenotypes [24,38]. Our attention was directed to the identification of FEN1 and PCNA in the immunoprecipitates since both proteins are known to be important in genetic stability [4,41]. Subsequent biochemical experiments with purified proteins demonstrated that FEN1/hWH are capable of associating with each other. There is no direct affinity between hWH and PCNA. The association of PCNA is likely through FEN1 since PCNA and FEN1 are known interacting partners [39] and such interactions have important functions in the maturation of precursors of nascent lagging strand near replication forks [3]. Interestingly, the presence of hWH at the site of DNA replication along with FEN1/PCNA is also supported by the co-localization results using immunofluorescence microscopy.

FEN1 is a structure-specific nuclease that can remove extrahelical 5′ DNA or RNA flaps; its function is essential for replication and repair [5]. While the presence of FEN1 at or near the replication forks is critical for the maturation of the newly synthesized Okazaki fragments, it also poses a potential threat for replication forks since FEN1 can cleave at the fork junctions where trihelical segments intersect, and generate double strand breaks [11]. Our biochemical result that hWH can differentially modulate FEN1’s activities depending on the structure of DNA substrate, suggests a plausible function of hWH in protecting genome stability. hWH is able to suppress FEN1’s endonucleolytic cleavage of fork and gap DNA substrates relative to its activities toward flap substrates, thereby minimizing the wayward activity of FEN1 while still maintaining its functionally important ability to process the nascent lagging strands. In our activity assays, we observed hWH modulating FEN1’s multiple nucleolytic activities with hWH concentrations up to 1 mM. In the intracellular environment, hWH/FEN1 may cooperate with other proteins as components in a macromolecular complex at the replication fork. Therefore there may be more effective interactions between hWH and FEN1 to regulate FEN1’s activities in the replication machine. Alternatively, hWH may possess post-translational modifications as yet to be characterized to enable hWH to interact more efficiently with FEN1. In contrast to other interacting proteins that can either promote or inhibit FEN1’s activities, hWH has diametric effects on the gap versus flap endonuclease activities, which are consistent with the proposed functions of hWH in protecting fork integrity. This proposed function of hWH in genome stability is further supported by the RNA knockdown experiments of hWH and FEN1. While the addition of siRNA for FEN1 results in negligible effects on cell viability and genome integrity, double knockdown of hWH and FEN1 by siRNA can partially mitigate loss of cell viability and DNA damage caused by hWH knockdown. This result thus suggests that FEN1 is at least partially responsible for DNA damage caused by reducing the intracellular WH levels.

WH is a member of an evolutionarily conserved family of proteins with multiple WD40 repeats, which has its homologs in yeast known as TRM82 and in mammals as WDR4 [21,24,47]. Proteins with WD40 repeats usually have a disk-like β-propeller structure with multiple blades composed of anti-parallel β-sheets [48]. WD40-repeat proteins are known mediators in the assembling of protein complexes important for various cellular functions [49]. Besides FEN1, WH’s homologs are known to interact with the catalytic subunit of tRNA methyl transferase, TRM8 in yeast and METTL1 in mammals [24,37]. The crystal structure of TRM8/TRM82 complex has been solved, revealing that TRM82 has a β-propeller structure of seven blades with the edges of two of the blades contacting TRM8 [22]. It is uncertain whether WH’s interaction with tRNA methylase has an important role in its genome guardian function. However since yeast mutants of *trm8* or *trm82* do not have any serious phenotypes and, as we have shown here that siRNA knockdown of METTL1 does not affect cell viability and genome integrity, tRNA methylation through WH’s function is likely not a major contributor to genome stability.

FEN1, on the other hand, is known to be an essential protein for DNA replication and repair, and has specific functions in removing RNA primer on nascent lagging strands near replication forks and in long patch base excision repair [5]. It therefore comes as no surprise that there are a number of proteins discovered to date that interact with FEN1, including PCNA, 9-1-1, Werner syndrome protein (WRN), and Bloom’s syndrome protein (BLM), and that these partner proteins can stimulate FEN1’s flap endonuclease activity about 5–11 fold [14,17,39,50,51]. Besides protein/protein interactions, FEN1 is also subject to multiple cell cycle-specific post-translational modifications [20]. Our results presented here demonstrated WH as a new interacting partner with FEN1 and that their association can modulate the structure-specific endonuclease activities of FEN1. Such regulatory functions of FEN1 may have a critical role in the growth and development of a multicellular organism and in the maintenance of its genome stability.

## Materials and Methods

### Ethics Statement

All the animals utilized in this study were maintained in a specific pathogen free environment under the guidelines of Academia Sinica Institutional Animal Care and Use Committee.

### Cell Lines

All mammalian cell lines including JB6 mouse epidermal cells, HFW human fibroblast cells, and HCT116 p53^+/+^ and HCT116 p53^-/-^ cells were maintained in Dulbecco’s Modified Eagle Medium (DMEM) supplemented with 10% heat-inactivated fetal bovine serum (FBS), 100 unit/ml penicillin, and 100 μg/ml streptomycin at 37°C in a humidified incubator with 5% CO_2_. *Drosophila melanogaster* Schneider 2 (S2) cells were maintained in Schneider’s medium supplemented with 10% heat-inactivated FBS, 100 units/ml penicillin, and 100 μg/ml streptomycin at 28°C. All reagents used in cell culture were from Invitrogen (Grand Island, NY, United States).

### Antibodies

We synthesized peptides LKKKRQRSPFPGSPEQTK from protein sequences of mouse Wuho and DGHAKKMRPGEATLSC from human Wuho, and used them to immunize rabbits. The antibodies were purified by affinity chromatography with peptide antigens before being used for western blot or immunofluorescence.

### RNA Interference (RNAi)

Small interference RNA (siRNA), both gene-specific ON-TARGET and controls Non-targeting pool siRNA, were purchased from Dharmacon (Chicago, IL, US), and used for knockdown experiments following manufacturer’s protocol. Single siRNAs against human or mouse FEN1 were purchase from Ambion (Grand Island, NY, US). The sequences for all the siRNA used in this work are listed in S1 Table.

### Viability Assay

Cellular viability was determined by MTT assay using a water-soluble reagent WST-8 (Dojindo, Kumamoto, Japan).

### Cell Assays

DNA laddering in apoptotic cells was used as a cell death assay. DNA fragments were purified by Suicide Track Kit (Millipore, Billerica, MA, US), and analyzed by electrophoresis in 2% agarose gels.

DNA double strand breaks were monitored by comet assay (single cell gel electrophoresis in neutral conditions) as described before [52]. Migration of fragmented DNA escaping from the nucleus in each cell was measured with the COMET Assay III program (Perspective Instruments, Suffolk, United Kingdom) and expressed by the parameter of the tail moment (a product of the tail length and intensity).

### Knockout of Mouse *Wh* (m*Wh*) Gene

Knockout of the m*Wh* gene was carried out by the Cre-loxP system with a targeting construct to delete exons 2 and 3. The backbone of the targeting vector, which harbors a m*Wh* genomic region including exon1, 2, and 3, was subcloned from a BAC clone RPCI23.C (Invitrogen) by BAC Subclone Kit (K003, GENE BRIDGES). The insertions of one loxP site between exon1 and 2 and one PGK-neo cassette (positive selection marker) flanked by two loxP sites behind exon3 were achieved by the recombineering method using Quick and Easy Conditional Knockout Kit (loxP/Cre) (K005, GENE BRIDGES). A PGK-DT (diphtheria toxin, negative selection marker) cassette was inserted into the vector at the *Eco*RV and *Sal*I sites. The resulting construct was linearized by *Sna*BI prior to electroporation into C57BL/6J ES cells. After screening by neomycin treatment, targeted ES cell clones were microinjected into C57BL/6-C2J blastocysts for generating chimera mice. The male chimera mice were then crossed with wild-type C57BL/6-C2J females. The black pups with targeted allele were crossed with EIIa-Cre mice [53] to generate m*Wh* heterozygous mice (+/-) carrying one m*Wh* null allele, through deleting exon2 and 3 between the first and the third loxP sites. Homozygous deletion mice (-/-) were generated by the intercross of +/- mice.

After marking littermates by ear notching, genomic DNA was isolated from ear tissue for genotyping. Genotyping was performed by PCR with two primers (ACCACGAGCCTAGAGGATCAGTGGC and TTGTCTGTCTGTGGGAGGGCCTGAG), which can identify wild-type and null alleles by amplifying 3.6 kb and 0.5 kb fragments, respectively.

### Identification of Human WH (hWH) Interacting Partners

A site-specific recombination system was used to generate a stable cell line of 293T with an integrated gene for h*wh* under a tetracycline-inducible promoter. A plasmid vector containing pcDNA5/FRT/TO (Invitrogen) was inserted with h*wh* cDNA flanked with V5 and hexahistidine epitope tags, and the resulting construct was used to transfect Flp-In-293 cells (Invitrogen) to allow for integration of h*wh* cDNA into the genomic FRT site. Clonal selection of stable recombinants was carried out with 300 μg/ml hygromycin and 10 μg/ml blasticidin in the media. Cells were induced with 1 μg/ml tetracycline for 24 h, and harvested cells were resuspended in a hypotonic buffer of 5 mM KPO_4_ (pH 7.8), 2 mM MgCl_2_, 1 mM EDTA, and protease inhibitor cocktail (Roche, Basel, Switzerland). Cells were lysed by douncing and nuclei were collected by centrifugation at 3 k*g* for 5 min. The nuclear pellet was suspended with nuclear extraction (NE) buffer containing 20 mM Hepes (pH 7.9), 400 mM NaCl, 10% glycerol, 1 mM EDTA, 0.2% NP-40, and protease inhibitor cocktail. The nuclear extract was incubated with 50 μl mouse anti-V5 agarose beads (Sigma, St. Louis, MO, US) with rotation at 4°C overnight. In parallel, nuclear extract of control Flp-In-293 cells transfected with vector DNA was processed under identical conditions. The beads were washed 5 times with NE buffer prior to boiling in SDS-PAGE sample buffer without any thiol reagents. The sample with eluted protein was replenished with 5% β-mercaptoethanol and boiled again before electrophoresis in a 4%–12% SDS-polyacrylamide gel. Following Coomassie Blue staining, protein bands were sliced and identified by Mass Spectrometry.

### Purification of Recombinant Human WH (hWH) and Flap Endonuclease 1 (FEN1)

FEN1 was purified using a published protocol [54] with a construct kindly provided by Dr. Robert Bambara. For purification of hWH, we made a vector with hWH cDNA inserted into pET23b (Novagen, Madison, WI, US) carrying a hexahistidine tag at its C-terminus, and transformed it into

BL21(DE3)pLysS. Expression of hWH was induced by 1 mM IPTG at 37°C for 4 h. The cell pellet was lysed with a buffer containing 20 mM Tris pH 8.0, 300 mM NaCl, 5 mM β-mercaptoethanol, 10% glycerol, 20 mM imidazole, and protease inhibitor cocktail. The soluble fraction was applied to the HisTrap FF Crude column (GE, Waukesha, WI, US), and hWH was eluted with 200 mM imidazole. The pooled factions were applied to a Hitrap Q FF column (GE), and eluted by a NaCl gradient from 0.05–1 M with hWH peaked at 0.3 M NaCl. The pooled fractions were dialyzed in a buffer containing 20 mM Tris pH 8.0, 0.3 M NaCl, 5 mM β-mercaptoethanol, and 50% glycerol, and stored at -30°C.

### Assays Monitoring hWH and FEN1 Interactions

For co-immunoprecipitation (co-IP) with cell lysates, HCT116 p53^+/+^ cells were lysed in RIPA buffer plus protease inhibitor cocktail. Cell lysate (1 mg/ml in protein) was pre-cleared by incubating with 50 μl of PureProteome Protein A Magnetic Beads (MILLIPORE) at 4°C for 1 h. The cleared lysates were mixed with magnetic beads conjugated with an antibody against target protein, either rabbit anti-hWH antibody or mouse anti-FEN1 antibody (abcam, Cambridge, UK), and incubated with 0.1 U/ml micrococcal nuclease at 4°C overnight. The partner proteins bound to beads were detected by western blots.

The direct pull-down assay was carried out with hWH, FEN1, and PCNA with a C-terminal tag of GST, hexahistidine and FLAG, respectively. To detect FEN1 binding with hWH by fluorescence anisotropy, we constructed an expression vector for a fusion protein of hFEN1 with a C-terminal tag of tetracysteine (FEN1-CCPGCC) [55]. The purified protein was incubated with 1mM TCEP for 30 min at 25°C prior to labeling with ReAsH reagent for 2 h at 25°C. The anisotropy of the fluorescence from FEN1-ReAsH was measured after adding different amounts of hWH; the binding data were used to determine the dissociation constant [56].

### Imaging with Fluorescence Microscopy

Prior to fixation with 4% paraformaldehyde, HCT116 p53^+/+^ cells were fed with a culture medium containing the thymidine analog, 5-ethynyl-2′-deoxyuridine (EdU) (Click-iT EdU Imaging Kit, Invitrogen), in 10 μM for 15 min. The fluorophore (Alexa488 azide) was linked to EdU by click reaction catalyzed by cuprous ions [57] marking the nascent DNA. Fixed cells were probed with rabbit anti-hWH, mouse anti-FEN1 (abcam), and goat anti-PCNA (Santa Cruz Biotechnology Inc., Santa Cruz, CA, US) antibodies. The fluorophore-conjugated secondary antibodies were conducted by incubation with Alexa555 anti-goat, Alexa594 anti-mouse, and Alexa647 anti-rabbit antibodies (Invitrogen). Images were collected with a TCS-SP5-AOBS-MP microscope (Leica, Wetzlar, Germany). The extent of image-overlaps was determined with Metamorph (Molecular Devices, Sunnyvale, CA, US).

We also used dSTORM (direct stochastic optical reconstruction microscopy) to map the EdU and hWH localization with super-resolution imaging [43]. Cells were pulse-labeled with EdU, and the fixed cells were labeled with rabbit anti-hWH antibody, followed by Cy5 anti-rabbit secondary antibody (Invitrogen). The samples were immersed in 100 mM mercaptoethylamine, PBS, before image collection by dSTORM.

### Preparation of Fluorophore-Labeled DNA Substrates

PAGE-purified DNA oligonucleotides with or without fluorophore of FAM or Cy5 were obtained from PURIGO (Taipei, Taiwan). Their sequences are listed in S2 Table. Oligonucleotides were adjusted to 100 μM with 10 mM Tris-HCl pH 8.5. The fluorophore-labeled oligonucleotide was annealed to unlabeled oligonucleotides (S3 Table) in 10 mM Tris-HCl pH 8.5, 100 mM KCl, and 5 mM MgCl_2_, with temperatures slowly ramping from 95°C to 25°C at a rate of 0.01°C/sec. Annealed products were separated by polyacrylamide gel electrophoresis in Tris-borate-EDTA buffer (TBE-PAGE). DNA substrates were eluted by immersing gel slices in 0.5 M ammonium acetate at 4°C with rotation overnight. The sample was applied to an Illustra NAP-5 column (GE) and eluted with 10 mM Tris-HCl pH 8.5 with 100 mM NaCl. The concentrations of fluorophore-labeled DNA substrates were measured by TBE-PAGE with fluorophore-labeled DNA oligonucleotides as standards. Design of oligonucleotides is based on a previous reference [58], with modifications.

### FEN1 Activity Assay

Fluorophore-labeled DNA substrates with concentration of 50 nM were incubated with FEN1 at 37°C for certain time (1 min for single-flap and double-flap, 5 min for nick, and 30 min for Y-shape and gap) in 20 μl reaction buffer containing 50 mM Tris (pH 8), 50 mM NaCl, and 5 mM MgCl_2_. Reactions were terminated by the addition of a 2x stop mix containing 95% formamide, 10 mM EDTA (pH 8), and 0.2% Orange G. Reaction products were denatured at 95°C for 5 min and separated by denaturing TBE-PAGE with 7M urea polyacrylamide gel and fluorescence images were captured and analyzed by ImageQuant LAS 4000 (GE). The assays on FEN1’s gap endonuclease activity in Y-shape and gap substrates were also examined by native TBE-PAGE.

### Electrophoretic Mobility Shift Assay

Fluorophore-labeled double flap substrate and Y-shape substrate of 50 nM was incubated with either FEN1 or hWH (concentrations ranging among 25, 50, 100, 250, 500, and 1,000 nM) at 37°C for 10 min in 20 μl binding buffer containing 50 mM Tris (pH 8) and 50 mM NaCl. Incubation products were separated by TBE-PAGE and images were processed by ImageQuant LAS 4000 (GE).

## Supporting Information

S1 Data Underlying data for Figs 1C, 1E, 4A, 4C, 4D, 6E, 8B, 9A, 9B, 10A, S1B, S1D, S5, S6A, S7A and S7B. (XLSX)

S1 Fig Knockdown of Wuho (WH) in *Drosophila* S2 cells induces DNA damage and apoptosis. (A) *Drosophila* WH was depleted by treatment of 25 nM of WH siRNA (siWH) in cultured cells for 72 h. No changes in WH levels were observed in Control (cells without treatment) or siControl (cells treated with control siRNA) groups. (B) Depletion of WH causes loss of viability as determined by MTT assays. (C) Depletion of WH induces DNA damage revealed by γ-H2AX staining. (D) DNA strand breaks monitored by neutral comet assay. (E) DNA laddering reveals apoptosis in cells with WH knockdown. (F) The apoptotic event was also observed by nuclear condensation stained by DAPI (indicated by arrows). Single asterisk and double asterisks indicate significant differences when compared with the control groups at *p* < 0.05 and *p* < 0.01 levels, respectively, according to Student’s *t* test.
(TIF)

S2 Fig Knockdown of mWH in mouse cells induces DNA damage and cell death. (A) Depletion of mWH induces apoptosis in mouse JB6 cells through a pathway identical to that in human cells by activating ATM, Chk2, and p53. (B) The apoptotic pathway also goes through activation of Caspases-9 and -3, but not Caspase-8, identical to that in human cells.
(TIF)

S3 Fig hWH interacts with PCNA through FEN1. (A) PCNA can interact with FEN1-His_6_ by pull-down assay with Nickel beads (lane 1), but not with hWH-His_6_ (lane 2). PCNA does not bind to Nickel beads in the control experiment (lane 3). (B) With pull-down experiments using GSH beads, hWH-GST can interact with PCNA only in the presence of FEN1 (lane 1), but not in its absence (lane 3). hWH-GST does not interact with PCNA directly (lane 3). The control experiments demonstrate the specificity of these experiments (lanes 4–6).
(TIF)

S4 Fig hWH’s function is not directly related to DNA repair process. (A) HCT116 p53^+/+^ cells were treated with X-ray irradiation of 2 or 20 Gy. After 1, 12, and 24 h, cells were harvested and processed to measure expression levels of γ-H2AX, hWH, and actin by western blot. (B) HCT116 p53^+/+^ cells were treated with 2Gy X-ray and subsequently stained with DAPI (blue), hWH (red), and γ-H2AX (green) for immunofluorescence microscopy.
(TIF)

S5 Fig Depletion of FEN1 mitigates DNA damage and cell death resulting from mWH knockdown in mouse cells. Double knockdowns of mWH and FEN1 were conducted in mouse JB6 cells by siRNAs. We utilized the siRNA pool for mWH knockdown, and 3 different single siRNAs for FEN1 knockdown. It is interesting to note that the siRNA least efficient in reducing FEN1 expression (simFEN1-2) was also least capable in rescuing the cytotoxic effect from mWH knockdown (lanes 3 and 7). The depletion efficiency of mWH and FEN1, and the DNA damage level (γ-H2AX) were determined by western blot. Viability of each treatment was measured by MTT assay.
(TIF)

S6 Fig tRNA methyltransferase function of hWH is not relevant for its role in genome stability. (A) Depletion of METTL1 (catalytic subunit of tRNA methyltransferase) in HCT116 p53^+/+^ cells by siRNAs does not induce DNA damage (monitored with γ-H2AX signals), nor cell death. (B) There are no significant co-localizations between EdU and METTL1 examined by confocal. Confocal images of HCT116 p53^+/+^ cells were stained to differentially mark nuclei (blue), EdU (green), and METTL1 (red).
(TIF)

S7 Fig hWH inhibits FEN1’s gap endonuclease activity on Gap (A) and Y-shape (B) substrates analyzed by TBE-PAGE under non-denaturing conditions. Structures of FAM (asterisk)-labeled gap substrate, and FAM /Cy5 (green and red asterisk, respectively) doubly labeled Y-shape (fork) substrate with cleavage sites marked by arrows are shown on the top. (A) In the gap substrate, cleavage product is marked with “a.” (B) In the fork substrate, products after cleavage at lagging strand are marked with “a” and “a;” those after leading strand cleavage are marked with “b.” The concentrations of DNA substrates were 50 nM, and those for FEN1 and hWH were indicated below. Single asterisk and double asterisks indicate significant differences at *p* < 0.05 and *p* < 0.01 levels, respectively, according to Student’s *t* test.
(TIF)

S8 Fig hWH does not bind to double flap and Y-shape DNA substrates. The affinities of FEN1 and hWH were monitored by electrophoretic mobility shift assay. Fluorophore-labeled DNA substrates were incubated with proteins with concentrations ranging among 25, 50, 100, 250, 500, and 1,000 nM. Products were examined by TBE-PAGE.
(TIF)

S1 Table Names and sequences of siRNAs targeted to specific genes used in this study. (XLSX)

S2 Table Sequences of oligonucleotides used in making DNA substrates. Oligonucleotides 1–3 have a FAM fluorophore at their 5′ end and oligonucleotide 4 has a Cy5 fluorophore at its 5′ end.
(XLSX)

S3 Table Component oligonucleotides in S3 Table used for each DNA substrate. (XLSX)

## Abbreviations

coIP: co-immunoprecipitation
dSTORM: direct stochastic optical reconstruction microscopy
EdU: 5-ethynyl-2′-deoxyuridine
FEN1: flap endonuclease 1
hWH: human Wuho protein
METTL1: methyltransferase-like protein 1
mWH: Mouse Wuho protein
PCNA: proliferating cell nuclear antigen
siRNA: small interference-RNA
TIRF: total internal reflection fluorescence
*wh*/WH: *wuho*/Wuho
