# Supplementary figures and images for: Wuho Is a New Member in Maintaining Genome Stability through its Interaction with Flap Endonuclease 1
Source: PLoS Biol. 2016 Jan 11;14(1):e1002349. doi: 10.1371/journal.pbio.1002349 (PMC4709127; doi:10.1371/journal.pbio.1002349)

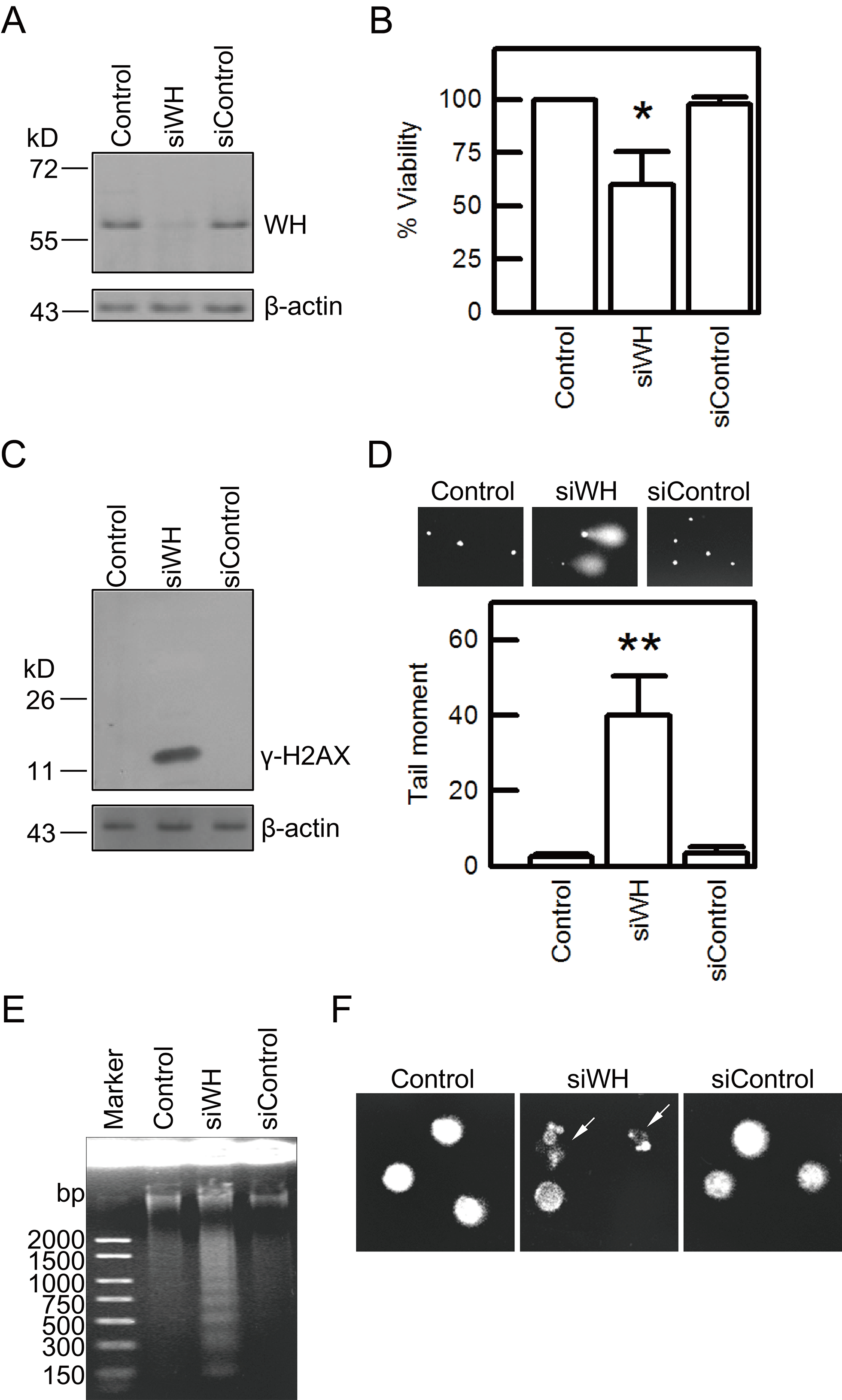

Supplement: S1 Fig — (A) Drosophila WH was depleted by treatment of 25 nM of WH siRNA (siWH) in cultured cells for 72 h. No changes in WH levels were observed in Control (cells without treatment) or siControl (cells treated with control siRNA) groups. (B) Depletion of WH causes loss of viability as determined by MTT assays. (C) Depletion of WH induces DNA damage revealed by γ-H2AX staining. (D) DNA strand breaks monitored by neutral comet assay. (E) DNA laddering reveals apoptosis in cells with WH knockdown. (F) The apoptotic event was also observed by nuclear condensation stained by DAPI (indicated by arrows). Single asterisk and double asterisks indicate significant differences when compared with the control groups at p < 0.05 and p < 0.01 levels, respectively, according to Student’s t test. (TIF) [file pbio.1002349.s002.tif]

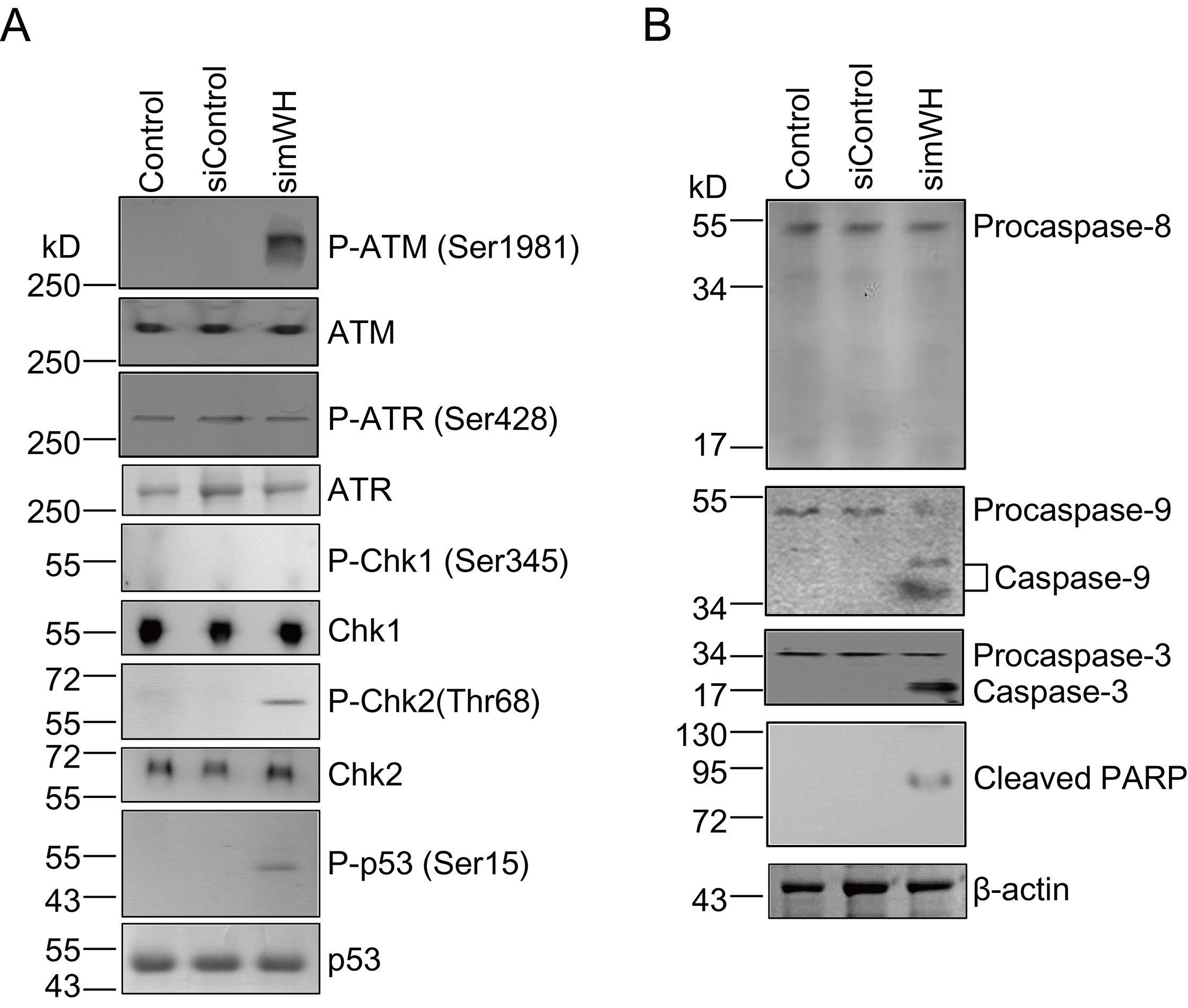

Supplement: S2 Fig — (A) Depletion of mWH induces apoptosis in mouse JB6 cells through a pathway identical to that in human cells by activating ATM, Chk2, and p53. (B) The apoptotic pathway also goes through activation of Caspases-9 and -3, but not Caspase-8, identical to that in human cells. (TIF) [file pbio.1002349.s003.tif]

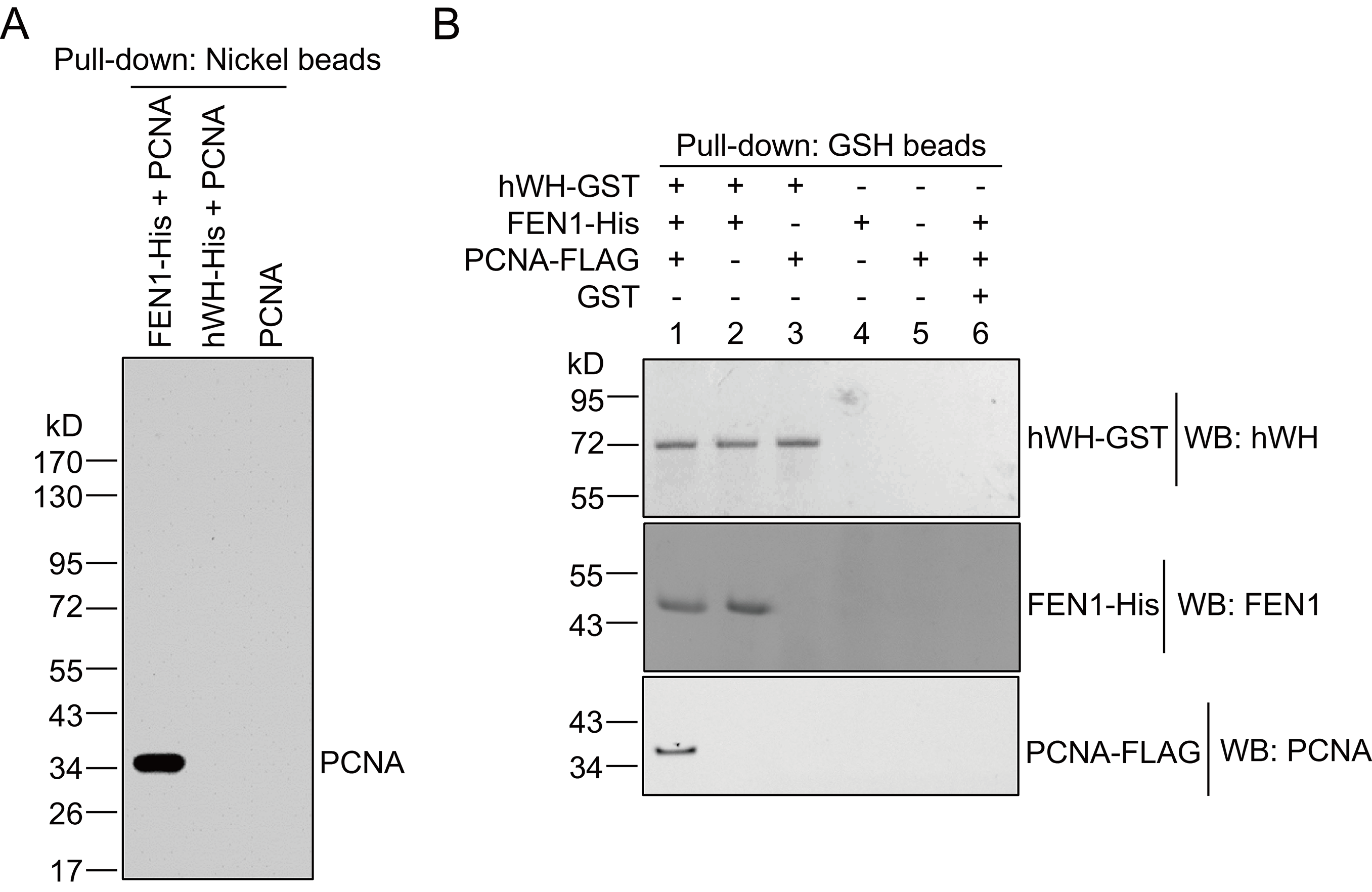

Supplement: S3 Fig — (A) PCNA can interact with FEN1-His6 by pull-down assay with Nickel beads (lane 1), but not with hWH-His6 (lane 2). PCNA does not bind to Nickel beads in the control experiment (lane 3). (B) With pull-down experiments using GSH beads, hWH-GST can interact with PCNA only in the presence of FEN1 (lane 1), but not in its absence (lane 3). hWH-GST does not interact with PCNA directly (lane 3). The control experiments demonstrate the specificity of these experiments (lanes 4–6). (TIF) [file pbio.1002349.s004.tif]

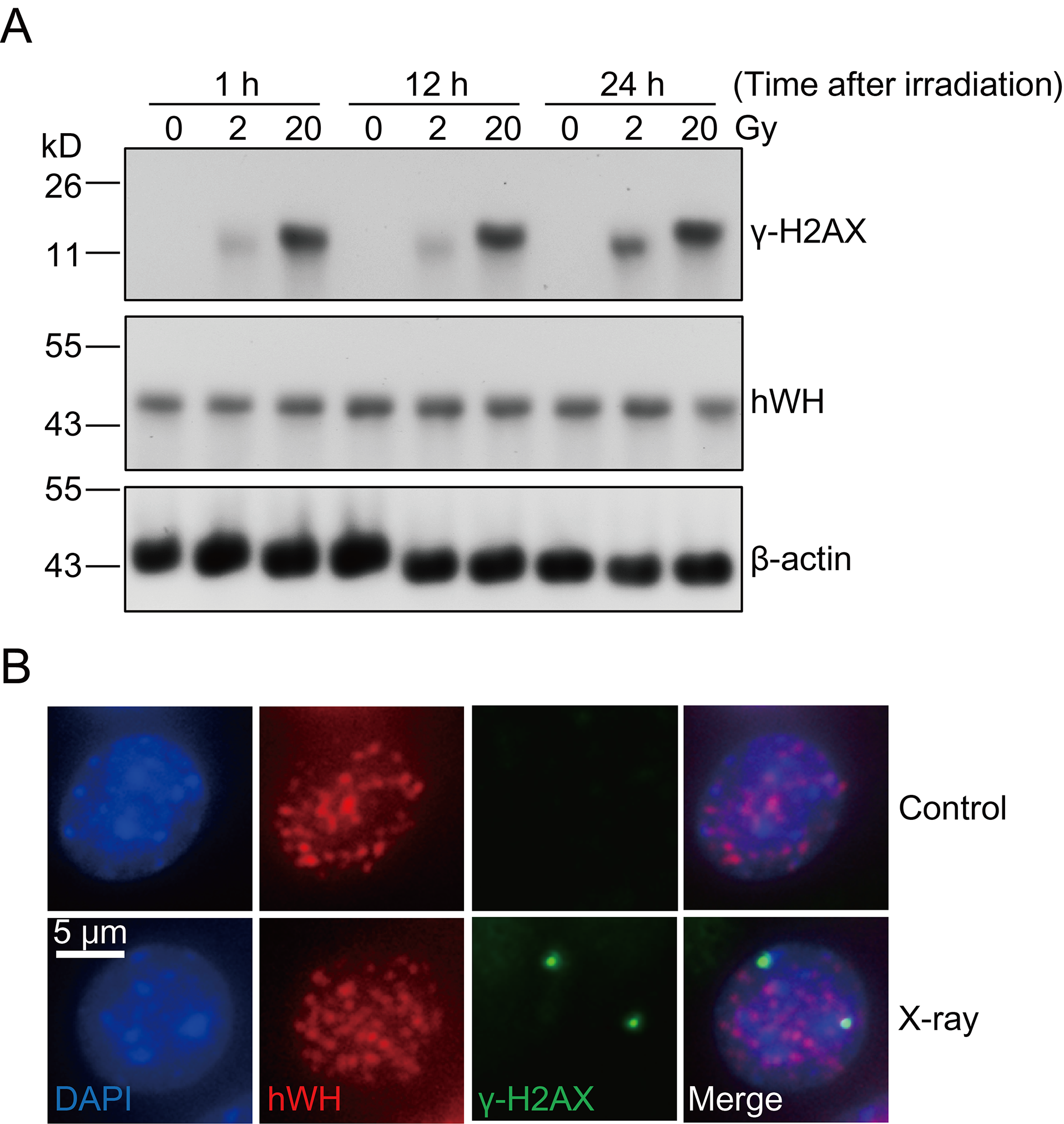

Supplement: S4 Fig — (A) HCT116 p53+/+ cells were treated with X-ray irradiation of 2 or 20 Gy. After 1, 12, and 24 h, cells were harvested and processed to measure expression levels of γ-H2AX, hWH, and actin by western blot. (B) HCT116 p53+/+ cells were treated with 2Gy X-ray and subsequently stained with DAPI (blue), hWH (red), and γ-H2AX (green) for immunofluorescence microscopy. (TIF) [file pbio.1002349.s005.tif]

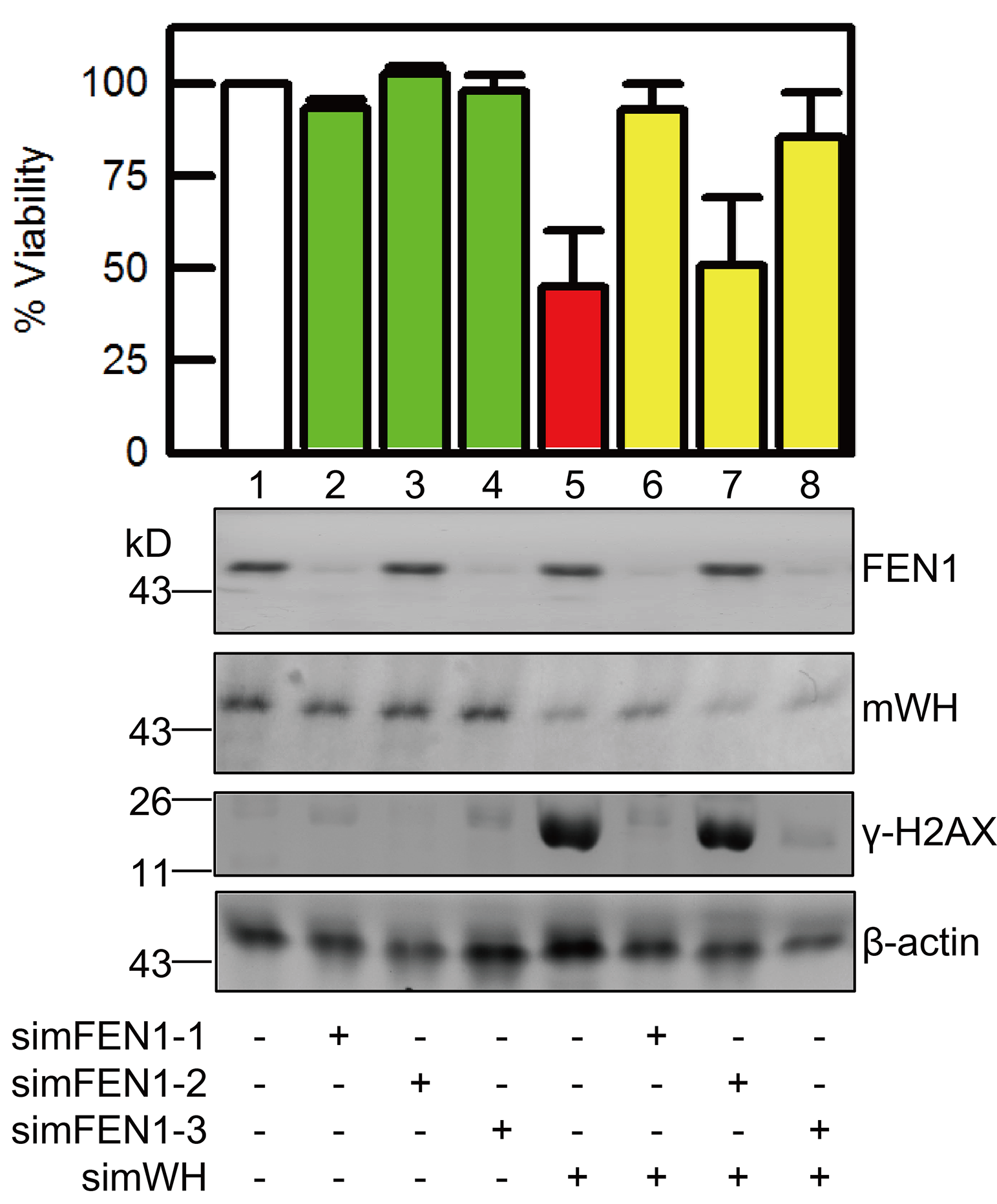

Supplement: S5 Fig — Double knockdowns of mWH and FEN1 were conducted in mouse JB6 cells by siRNAs. We utilized the siRNA pool for mWH knockdown, and 3 different single siRNAs for FEN1 knockdown. It is interesting to note that the siRNA least efficient in reducing FEN1 expression (simFEN1-2) was also least capable in rescuing the cytotoxic effect from mWH knockdown (lanes 3 and 7). The depletion efficiency of mWH and FEN1, and the DNA damage level (γ-H2AX) were determined by western blot. Viability of each treatment was measured by MTT assay. (TIF) [file pbio.1002349.s006.tif]

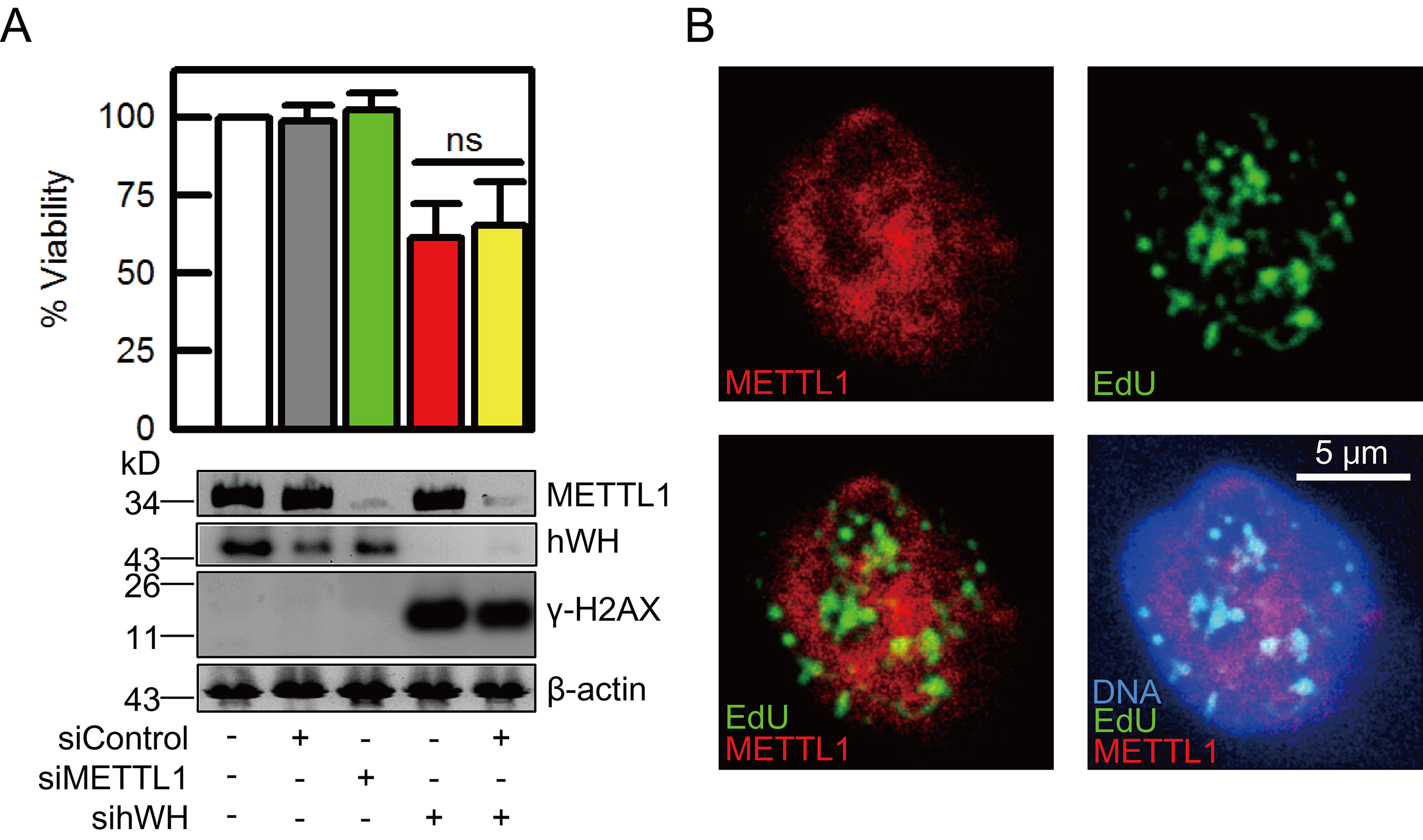

Supplement: S6 Fig — (A) Depletion of METTL1 (catalytic subunit of tRNA methyltransferase) in HCT116 p53+/+ cells by siRNAs does not induce DNA damage (monitored with γ-H2AX signals), nor cell death. (B) There are no significant co-localizations between EdU and METTL1 examined by confocal. Confocal images of HCT116 p53+/+ cells were stained to differentially mark nuclei (blue), EdU (green), and METTL1 (red). (TIF) [file pbio.1002349.s007.tif]

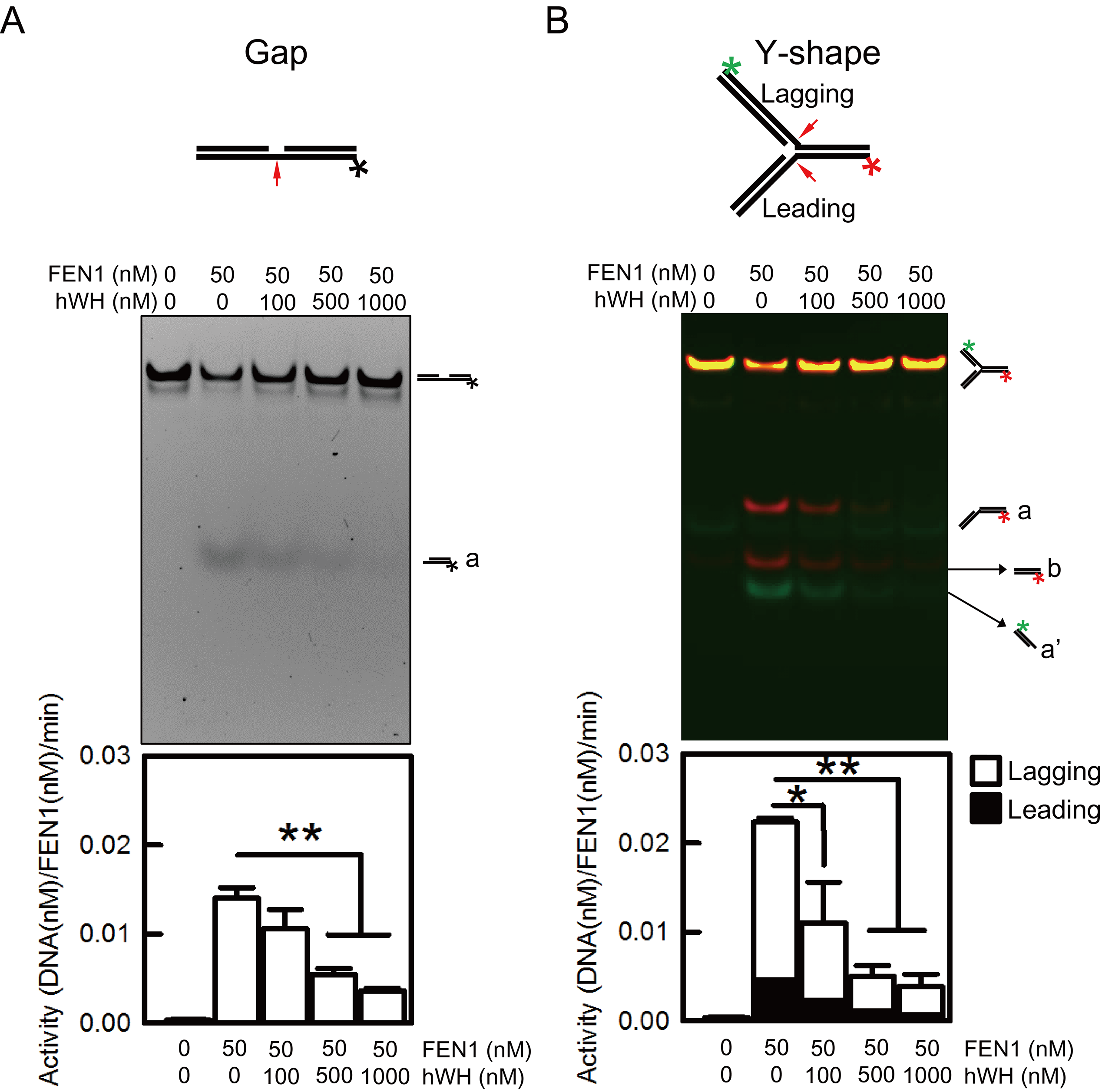

Supplement: S7 Fig — (A) In the gap substrate, cleavage product is marked with “a.” (B) In the fork substrate, products after cleavage at lagging strand are marked with “a” and “a;” those after leading strand cleavage are marked with “b.” The concentrations of DNA substrates were 50 nM, and those for FEN1 and hWH were indicated below. Single asterisk and double asterisks indicate significant differences at p < 0.05 and p < 0.01 levels, respectively, according to Student’s t test. (TIF) [file pbio.1002349.s008.tif]

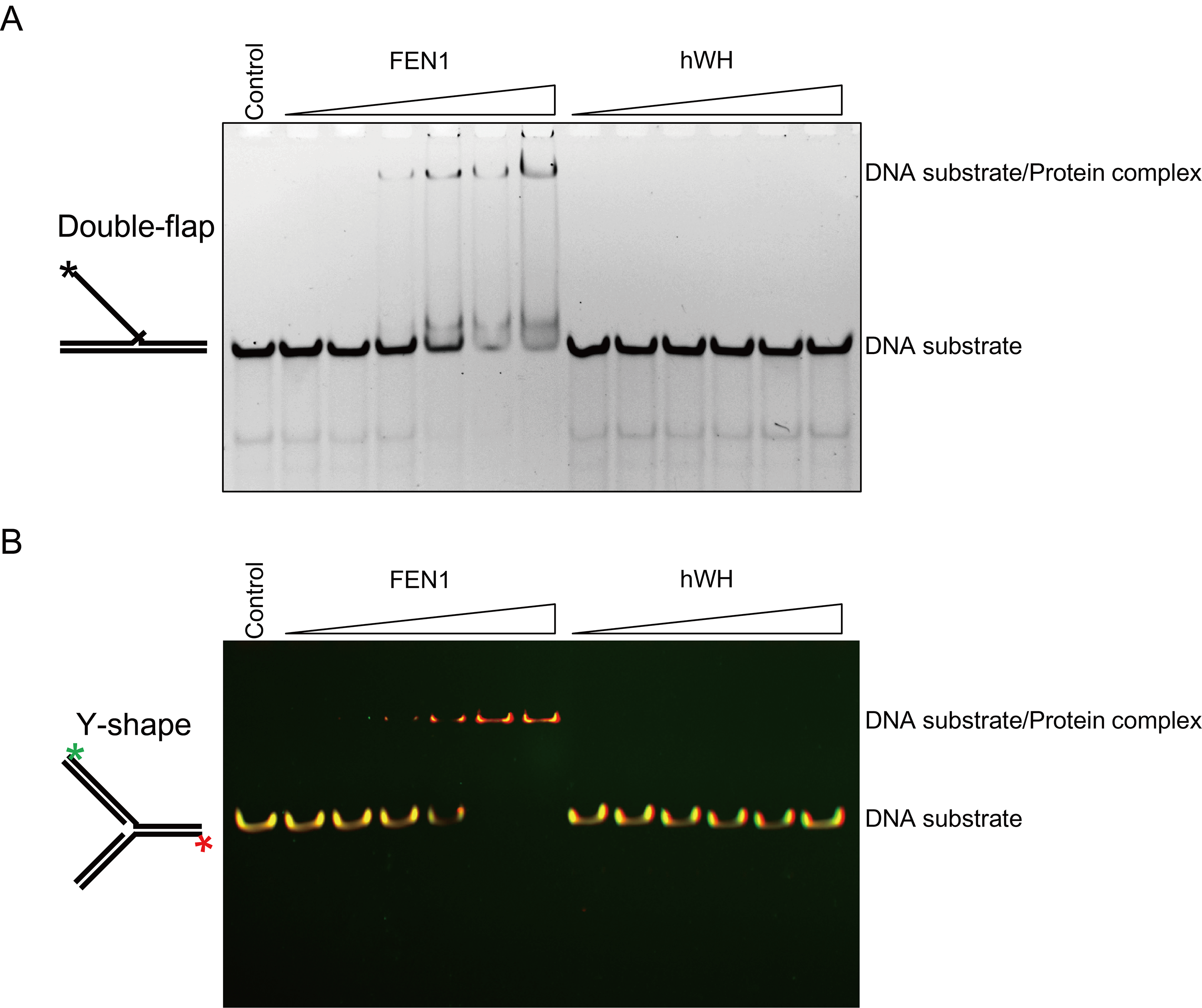

Supplement: S8 Fig — The affinities of FEN1 and hWH were monitored by electrophoretic mobility shift assay. Fluorophore-labeled DNA substrates were incubated with proteins with concentrations ranging among 25, 50, 100, 250, 500, and 1,000 nM. Products were examined by TBE-PAGE. (TIF) [file pbio.1002349.s009.tif]
